# How Well Do “Dr. Google” and “Professor YouTube” Answer Health Questions for Pediatric Kidney Transplant Recipients and Caregivers?

**DOI:** 10.1111/petr.70409

**Published:** 2026-08-11

**Authors:** Ashley Burghall, Ryan R. D. Chan, Nicola Rosaasen, Tom D. Blydt‐Hansen, Laura Beresford, Allison Cammer, Keefe Davis, Kayla Flood, Aviva Goldberg, Lorraine Hamiwka, Kathryn Haubrich, Camille Laroche, Véronique Phan, Michelle Ruhl, Chia Wei Teoh, Jenny Wichart, Holly Mansell

**Affiliations:** ^1^ College of Pharmacy and Nutrition University of Saskatchewan Saskatoon Saskatchewan Canada; ^2^ Division of Nephrology, Department of Pediatrics University of British Columbia Vancouver British Columbia Canada; ^3^ Department of Pharmacy Children's & Women's Health Centre of British Columbia Vancouver British Columbia Canada; ^4^ Division of Pediatric Nephrology, Department of Pediatrics Jim Pattison Children's Hospital, University of Saskatchewan Saskatoon Saskatchewan Canada; ^5^ Section of Nephrology, Department of Pediatrics and Child Health Children's Hospital, HSC Winnipeg Manitoba Canada; ^6^ Max Rady College of Medicine University of Manitoba Winnipeg Manitoba Canada; ^7^ Section of Nephrology, Department of Pediatrics, Cumming School of Medicine University of Calgary Calgary Alberta Canada; ^8^ Division of Nephrology, Department of Paediatrics, CHU Ste Justine Université de Montréal Montréal Quebec Canada; ^9^ Division of Nephrology, Department of Pediatrics Stollery Children's Hospital, University of Alberta Edmonton Alberta Canada; ^10^ Division of Nephrology, Department of Paediatrics The Hospital for Sick Children, University of Toronto Toronto Ontario Canada; ^11^ Department of Pharmacy Alberta Health Services Calgary Alberta Canada

**Keywords:** caregivers, Google, internet, patient education, pediatrics, YouTube

## Abstract

**Background:**

Pediatric kidney transplant recipients and their caregivers require comprehensive education to support self‐management. Increasingly, families supplement clinic teaching with online platforms such as Google and YouTube, yet the quality, readability, and usefulness of these resources are uncertain.

**Methods:**

Structured searches on Google and YouTube were conducted using patient‐style questions and medically oriented key terms across four prioritized topics: healthy eating, exercise, travel, and mental health. Resources were screened for relevance and assessed for accuracy, completeness (defined as including all key facts needed to support recommendations), readability, and actionability (defined as providing clear, practical steps users can follow). Two pediatric transplant healthcare providers independently reviewed each resource. Two researchers assessed understandability and actionability using the Patient Education Materials Assessment Tool (PEMAT), and readability using multiple readability tools.

**Results:**

Searches yielded 87 Google and 41 YouTube resources; most targeted adult transplant recipients. Healthcare providers assessed 65% of Google and 68% of YouTube resources to be accurate, but only 18% and 5% were factually complete. Actionable recommendations were identified in 42% (Google) and 29% (YouTube). Readability ranged from grade 5 to 17, with only 6 of 82 Google resources meeting the recommended grade 6 or lower. Mean understandability scores were 67% for both platforms. Actionability scores averaged 39% (Google) and 77% (YouTube). Resources suitable for pediatric audiences (*n* = 28) had similar readability and understandability scores but slightly higher actionability (Google: 43%, YouTube: 81%). Most resources lacked pediatric focus, co‐creation, and practical guidance.

**Conclusions:**

Online searches generated limited high‐quality resources tailored to pediatric needs. Healthcare providers should guide families to vetted resources and consider co‐creating materials to improve relevance and impact.

## Graphical abstract

Google and YouTube searches revealed few pediatric‐appropriate transplant resources, with most materials lacking completeness and actionable guidance. Findings highlight the need for clearer, more reliable online education for pediatric kidney transplant recipients and caregivers.

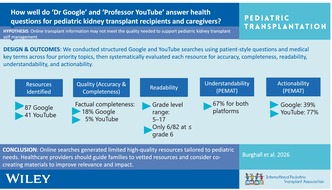

**Abbreviations**
CST: Canadian Society of Transplantation
PEMAT: Patient Education Materials Assessment Tool
PEMAT‐AV: Patient Education Materials Assessment Tool, Audio Visual
PEMAT‐P: Patient Education Materials Assessment Tool, Print

## 1 Introduction

Pediatric kidney transplant recipients and their caregivers must navigate complex, lifelong self‐management responsibilities, including medication adherence, infection prevention, healthy lifestyle behaviors, and the recognition of early signs of complications [1, 2]. Although multidisciplinary transplant teams provide structured education through the pre‐ and post‐transplant journey, families frequently supplement this information with online resources [3, 4, 5, 6, 7, 8]. Platforms such as Google and YouTube are among the most used sources of health information due to their accessibility, familiarity, and breadth of content. However, the quality, readability, and practical usefulness of these online materials vary widely, and their suitability for pediatric audiences is largely unknown. It is also unknown to what extent existing materials reflect patient or caregiver input, which may influence relevance and usability.

Emerging literature from other medical fields suggests that online health information is often incomplete, written at reading levels exceeding recommended standards, or lacks clear and actionable guidance that patients and caregivers can apply in daily life [9, 10, 11, 12]. Concerningly, patients and caregivers report difficulty evaluating the quality of online health information [6, 13]. Patients and caregivers often enact behavior changes based on online health information and infrequently discuss this information with their healthcare providers despite these quality concerns [8, 14, 15].

In response to these educational gaps, the Pediatric Educational Initiative (PEI) of the Canadian Society of Transplantation (CST) identified priority educational topics for pediatric transplant recipients and caregivers. Using these priorities as a foundation, the present study systematically evaluated the quality, readability, understandability, and actionability of online resources identified through Google and YouTube searches for four key topics prioritized by pediatric patients and families. Our objective was to determine how well current online resources support pediatric transplant self‐management and to identify gaps that may guide clinicians in directing families to trustworthy materials and inform the co‐creation of future patient‐centered resources.

## 2 Methods

We searched Google and YouTube to identify educational materials relevant for pediatric kidney transplant patients and caregivers. The resources were downloaded, and descriptive data was extracted into a data collection table. The resources were reviewed for accuracy, readability, and actionability.

### 2.1 Topic Selection

The PEI with the CST is a working group of healthcare professionals and patients formed to achieve consensus on important topics in pediatric transplant education. Through discussion and consensus building, the committee devised a list of 25 educational topics important for patients and caregivers to maintain a successful transplant. These topics include dental care, immunosuppressants, skin care, fluid requirements, and infection risks among others (Appendix S1). To narrow the scope of the present project, a patient and caregiver advisor (CJ and CK) selected four of the CST PEI's 25 education topics to be used in this study. These topics were chosen because our patient/caregiver advisor felt these were topics they were most likely to search online for information. These included: (1) Healthy eating, (2) Exercise, (3) Travel, and (4) Mental health.

### 2.2 Search Strategy for Obtaining Educational Materials

A preliminary search conducted by the research team (AB) identified that search results varied with the search term or phrase used. Previous work in other chronic diseases has shown that caregivers and patients seek out health information on search engines such as Google using short phrases [5, 6, 16]. Therefore, to simulate how pediatric transplant patients and their families search for information on pediatric kidney transplantation, the caregiver advisor devised a short search phrase for each study topic. To explore the difference in search results with variation in language, the research team developed a distinct set of search terms using standard medical terminology for the study topics. A complete list of the search terms and phrases used is available in Table 1.

**TABLE 1:** Search phrase and key terms used for Google and YouTube searches.

Topic	Search phrase	Key terms
Healthy eating	‘Are there any dietary restrictions for my child after their kidney transplant?’	Pediatric kidney transplant diet
Exercise	‘Are there any physical activities my child shouldn't do after their kidney transplant?’	Pediatric kidney transplant exercise limitations
Travel	‘What should I know about traveling with my child after their kidney transplant?’	Pediatric kidney transplant travel
Mental health	‘How do I take care of my child's mental health after their kidney transplant?’	Pediatric kidney transplant mental health

**FIGURE 1:**
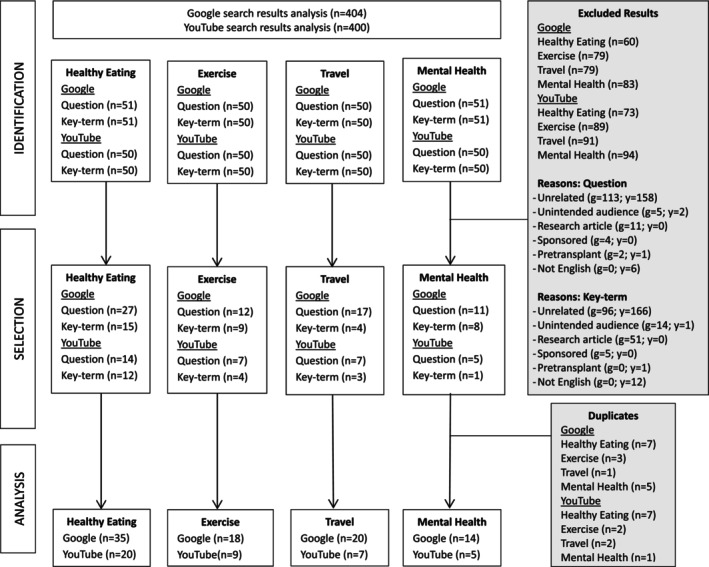
Search strategy.

Searches were conducted on Google (google.com) and YouTube (youtube.com) by two authors (A.B. and R.R.D.C.) on November 10, 2023, using the default settings and Incognito Mode on the Google Chrome web browser to minimize the influence of user data on search results. Studies assessing how internet users access online health information found that users rarely go beyond the first page of search results [16, 17]. However, to ensure that all relevant resources were identified, the first 50 search results on both Google and YouTube were reviewed. Educational resources were eligible for analysis if they were accessible within one click of the linked page, provided information directed towards solid‐organ transplant patients and/or caregivers, were open access, and available in English. Search results containing only transplant center specific facts such as contact information and location were excluded, as were academic papers intended for healthcare professionals and patient testimonials with no educational intent. YouTube Shorts, a type of short‐form video content available on YouTube, were also excluded. Two members of the research team (A.B. and R.R.D.C.) reviewed all search results for eligibility to ensure consensus. Discrepancies were resolved by a third reviewer (H.M.). The search strategy is provided in Figure 1.

### 2.3 Data Extraction

The source of the video/resource (YouTube or Google), format (e.g., website, online booklet, video), type of organization or persons that produced the resource (e.g., hospital/healthcare, industry‐sponsored, academic, government, independent users), country of origin, evidence of patient involvement (i.e., the resource was created by a patient or it was explicitly stated patients were involved), number of views/likes (as applicable), and CST PEI topic described was also recorded. Extracted data were organized into tables. The educational resources were then assessed for accuracy, readability, and actionability through healthcare provider reviews and standardized assessments for readability and actionability.

### 2.4 Healthcare Provider Reviews

A panel of healthcare providers consisting of pediatric transplant nephrologists (*n* = 9) and transplant pharmacists (*n* = 4) reviewed the resources. They were asked to comment on whether they would recommend the resource to their patients and caregivers with additional prompts to explore why or why not.

The reviewer form was drafted by two members of the research team (H.M. and T.D.B.‐H.), piloted on another team member (N.R.), and revised accordingly. The data collection tool consists of two questions assessing whether the healthcare provider would recommend the resource to a caregiver and/or pediatric transplant recipient, and five questions pertaining to accuracy, comprehensiveness, and actionability of the factual content and recommendations. Each resource was independently reviewed by two reviewers. (Table 2).

**TABLE 2:** Healthcare provider review form.

Questions	Response choices		Additional notes
1. Would you recommend the resource to a caregiver?	☐ Yes ☐ No		
2. Would you recommend it to a pediatric transplant recipient?	☐ Yes ☐ No	If yes, what age? ☐ ≤ 6 (grade 1 or lower) ☐ 7–10 (primary school) ☐ 11–13 (middle school) ☐ ≥ 14 (high school)	Choose all that apply
3. Is the factual content accurate?	☐ Yes ☐ No	If no, give examples of incorrect facts	If even ONE important fact is incorrect or misleading, please answer “No” and provide example(s)
4. Are the recommendations accurate?	☐ Yes ☐ No	If no, what was misleading/inaccurate	If even ONE recommendation is incorrect or misleading, please answer “No” and provide example(s)
5. Is the factual content complete?	☐ Yes ☐ No	If no, provide examples	‘Factually complete’ means that the resource includes ALL important facts relevant to the subject matter. An important fact is essential to support/explain a recommendation. If even ONE impt fact is missing, answer “No” and provide example
6. Are the recommendations complete?	☐ Yes ☐ No	If no, provide examples	Are there important clinically significant recommendations that are missing? Are there recommendations present that are not supported by facts described in the resource? If even ONE impt recommendation is missing, answer “No” and provide example
7. Are the recommendations actionable?	☐ Yes ☐ No	If no, provide examples	Are they specific and practical enough to be useful and achievable (The elements of a good recommendation include “what” to do and “how” to do it.) If even ONE key recommendation is not specific to transplant patients or does not include ‘what’ or ‘how’ to do what is recommended, please answer “No” and provide example(s)

### 2.5 Readability

Readability is an objective measure of the reading skills needed to comprehend a given text and are frequently described in “grade levels” [18]. Patient education materials that exceed an individual's reading skills, can lead to poor comprehension and negative outcomes. Given the lack of a validated tool for assessing the readability of patient education materials, the use of multiple readability tools is recommended to increase the validity of the results [18]. We used seven readability checkers available online [19] to assess eligible written resources: Flesch Reading Ease formula (FRE) [20], Gunning Index (FOG) [21], Automated Readability Index (ARI) [22], Flesch–Kincaid Grade Level (FKGL) [23], Coleman‐Liau Index (CLI) [24], SMOG Index (SMOG) [25], Linsear Write Formula (LW) [26], and Forecast Formula [27] and Average Reading Level Consensus Calculator [19]. Each tool assigns a score that correlates with the grade level or reading difficulty of the text based on average sentence, word length, and number of syllables. A grade level of five, for example, indicates that the average fifth grader can understand the material. (Supporting Information S1).

The relevant section from each printed resource was copied into an online readability calculator [19]. The online readability calculator calculates a score for each of the seven readability tools described above and assigns the text an overall score based on the average of the seven readability tools. Readability analysis was conducted for only objective 1 (Google search), since this search generated materials in print.

### 2.6 Understandability/Actionability

According to the National Action Plan to Improve Health Literacy, patient education materials must be accurate, accessible, and actionable [28]. Actionable patient educational materials help patients/caregivers with varying levels of health literacy in understanding what to do with or how to act on the presented material [29]. As pediatric patients and caregivers use online health information to assist with healthcare‐related decisions, the actionability of online resources is important [4, 8].

The actionability sub‐scale of the Patient Education Materials Assessment Tool (PEMAT) was used to evaluate the resources [30]. This tool consists of two versions: the PEMAT‐P and PEMAT‐AV, which are intended for printed and audio‐visual materials, respectively. The appropriate tool was selected depending on the nature of the resource. Each item was given a score of 0 (disagree) or 1 (agree) [31, 32]. The PEMAT‐P actionability subscale consists of 7 items, while PEMAT‐AV consists of 4. Scores for each resource were summed and divided by the maximum score to produce a percentage, with higher percentages indicating more actionable educational resources. Two reviewers (AB and RRDC) received training on the PEMAT tool and independently assessed each resource using the appropriate version. Discrepancies were resolved by a third reviewer (HM) (Supporting Information S2 and S3).

## 3 Results

### 3.1 Objective 1 (Google Search)

The top 50 Google search results for each topic (Healthy Eating, Exercise, Travel, Mental Health) and search strategy (question, key term) were evaluated by the research team. An additional four resources/results accessible within one click of a linked Google search result were also evaluated (*n* = 404). Following duplicate removal (*n* = 53), 87 unique educational resources were eligible for analysis (Healthy Eating: *n* = 35, Exercise: *n* = 18, Travel: *n* = 20, Mental Health: *n* = 14) (Supporting Information S4).

The format of the resources was mostly website‐based (*n* = 56). Additional formats included downloadable portable document format (PDFs) or booklets (*n* = 26) and video‐based content (YouTube videos, *n* = 4; other, *n* = 1). These resources were created by organizations from the United States (*n* = 55), Canada (*n* = 17), the United Kingdom (*n* = 9), Australia (*n* = 1), India (*n* = 1), Singapore (*n* = 1), and Istanbul (*n* = 1); or were multi‐site (United Arab Emirates/USA; *n* = 2). Most resources were created by hospitals/hospital groups for their own patients (*n* = 56), healthcare organizations (e.g., non‐profit, advocacy group; *n* = 21), or government agencies (e.g., health regions; *n* = 8). One resource was created by independent users (i.e., healthcare providers) and one resource was created by a drug manufacturer.

Most resources that met the inclusion criteria were directed towards adult transplant patients (*n* = 53). Of those pertaining to pediatric transplant education (*n* = 34), nineteen resources were directed towards caregivers, eight towards patients, and seven towards both patients and caregivers. Only eight of the resources eligible for analysis showed evidence of patient involvement in its creation (The top 3 resources are presented in Table 3, Supporting Information S5 provides full details).

**TABLE 3:** Google search results by topic.

#	URL/Reference	Search phrase	Title, authors, date	Format of resource	Type of organization producing resource	Country of origin	Target audience	Patient involvement
Healthy eating								
1	https://www.ucsfbenioffchildrens.org/education/nutrition‐after‐kidney‐transplant#:~:text=Choose%20low%2Dfat%20and%20low,activities%20are%20allowed%20after%20transplant.	Question, keyterm	Nutrition After Kidney Transplant, UCSF Benioff Children's Hospital medical specialists, unknown	Website	Hospital	USA	Caregivers (of Pediatric patients)	No
2	https://www.kidney.org/atoz/content/foods‐avoid‐after‐transplantation	Question	Foods to Avoid After Transplantation, National Kidney Foundation, 04/19	Website	Healthcare Organization	USA	Patients (Adult)	No
3	https://www.med.umich.edu/mott/pdf/transplant/pediatric‐kidney‐transplant‐diet‐nurtrition.pdf	Question, keyterm	Kidney Transplant Nutrition Therapy, C.S Mott Children's Hospital, unknown	Online booklet	Hospital	USA	Caregivers (of Pediatric patients)	No
Exercise								
1	https://www.aboutkidshealth.ca/Article?contentid=3952&language=English&hub=transplant#:~:text=Your%20child%20should%20avoid%20heavy,house%20or%20playing%20active%20games.	Question	Physical activity and sport post kidney transplant, Sickkids Staff (SickKids Hospital), 5/12/2021	Website	Hospital	Canada	Caregivers (Pediatric)	No
2	https://teens.aboutkidshealth.ca/Article?contentid=2699&language=English	Question, keyterm	Physical activity after a kidney transplant, SickKids Staff (SickKids Hospital), 11/30/2017	Website	Hospital	Canada	Patients (Pediatric)	No
3	https://www.youtube.com/watch?v=FL_E4vvRP0M&t=2s	Question	Transplant—Session 9: Lifestyle and Exercise, AboutKidsHealth—The Hospital for Sick Children, 4/03/2018	YouTube Video	Hospital	Canada	Patients (Pediatric)	Yes
Travel								
1	https://www.kidney.org/atoz/content/traveltip	Question	Travel Tips: A Guide for Kidney Patients, National Kidney Foundation, unknown	Website	Healthcare Organization	USA	Patients (Adult)	No
2	https://columbiasurgery.org/kidney‐transplant/resuming‐life‐after‐kidney‐transplantation	Question	Resuming Life After Kidney Transplantation, Columbia University Irving Medical Centre, unknown	Website	Hospital	USA	Patients (Adult)	No
3	https://www.kidneyfund.org/kidney‐donation‐and‐transplant/life‐after‐transplant‐rejection‐prevention‐and‐healthy‐tips/recovery‐after‐transplant‐surgery	Question	Recovery after transplant surgery, American Kidney Fund's Medical Advisory Committee, 02/16/2022	Website	Healthcare Organization	USA	Patients (Adult)	No
Mental health								
1	https://www.kidneyfund.org/kidney‐donation‐and‐transplant/life‐after‐transplant‐rejection‐prevention‐and‐healthy‐tips/mental‐health‐and‐support‐after‐transplant	Question, keyterm	Mental health and support after transplant, American Kidney Fund's Medical Advisory Committe, 2/16/2022	Website	Healthcare Organization	USA	Patient (Adult)	No
2	https://www.kidney.org/atoz/content/immunosuppression	Question, keyterm	Care after kidney transplant, National Kidney Foundation, unknown	Website	Healthcare Organization	USA	Patient (Adult)	No
3	https://kidshealth.org/en/parents/kidney‐transplant.html	Question	Kidney Transplant (for Parents), Kidney Transplant Program at Nemours Children's Health, 11/2022	Website	Hospital (Group)	USA	Caregiver (Pediatric)	No

### 3.2 Objective 2 (YouTube Search)

The first 50 YouTube search results for the topics: Healthy Eating, Exercise, Travel, and Mental Health using each search strategy (question, key term) were evaluated by the research team. Four‐hundred videos were analyzed, and 41 unique videos (Healthy Eating: *n* = 20, Exercise: *n* = 9, Travel: *n* = 7, Mental Health: *n* = 5) met the inclusion criteria (Figure 1).

Most videos were created by organizations/individuals from the United States (*n* = 31) and India (*n* = 9). One video was created by a Canadian organization. Similar to Objective 1, most resources were directed towards adult patients (*n* = 34). Three videos were directed towards pediatric caregivers, two resources were directed towards adult patients and their caregivers, and two resources were directed towards both pediatric and adult audiences; one video was directed to patients, and one was to patients and caregivers. Compared to the resources identified via a Google Search, the resources on YouTube were more likely to involve patients in their creation (*n* = 9) (The top 3 resources are presented in Table 4, Supporting Information S6 provides full details).

**TABLE 4:** YouTube search results by topic.

#	URL/Reference	Search phrase	Title, authors, date	Type of organization producing resource	Country of origin	Target audience	Evidence of patient involvement	Number of video views	Number of likes
Healthy eating									
1	https://youtu.be/PKUXMOM6ZrE?si=‐Z57y2PKEYv_‐wSy	Question, keyterm	Nutrition After Kidney Transplant, UCSF Benioff Children's Hospital medical specialists, unknown	Independent (Healthcare Provider)	USA	Patient (Adult)	No	25 017	667
2	https://youtu.be/ShQNrFfEYYs?si=ILU_b3x879gxnLmM	Question, keyterm	Kidney Transplant and What Foods to Eat and Avoid After, Blake Shusterman aka The Cooking Docs, 2022‐Jul‐25	Hospital	USA	Patient (Adult)	No	28 800	204
3	https://youtu.be/_QEyo00APpA?si=9LtsDN8sd_nI‐o8e	Question	What You Should and Shouldn't Eat or Drink After Your Kidney Transplant, Cleveland Clinic, 2019‐Nov‐04	Hospital	USA	Patient (Adult)	No	16 626	129
Exercise									
1	https://youtu.be/_QEyo00APpA?si=U_f4gz2lzs5imkBf	Question, keyterm	6 Things You Should Know About Life After Kidney Transplant Surgery, Crozer Health, 2018‐Nov‐15	Hospital	United States	Patient (Adult)	No	16 629	129
2	https://youtu.be/e2‐7Cp3KeLI?si=Q1BkRe7B6B59qrdg	Question	Care for Your New Kidney: What You Need to Know After Your Kidney Transplant, Cleveland Clinic, 2022‐Dec‐13	Hospital	United States	Patient (Adult)	No	949	23
3	https://youtu.be/yuEi5Yf5nR8?si=eL972fyRLiCxPjrb	Question	Can competitive athletics continue after a Kidney Transplant?, Sankaran Sundar (MD), 2017‐Jul‐28	Independent (Healthcare Providers)	India	Patient (Adult)	No	1957	23
Travel									
1	https://www.youtube.com/watch?v=F_zv‐0KxAuk&pp=ygVRV2hhdCBzaG91bGQgSSBrbm93IGFib3V0IHRyYXZlbGxpbmcgd2l0aCBteSBjaGlsZCBhZnRlciB0aGVpciBraWRuZXkgdHJhbnNwbGFudD8g	Question, keyterm	Traveling After A Kidney Transplant…MUST WATCH!!! | Travel tips for kidney patients, Crissy Mac, 2022‐Oct‐19	Independent (patient)	United States	Patient (Adult)	Yes	762	31
2	https://www.youtube.com/watch?v=v5ASWIAT1s4&pp=ygVRV2hhdCBzaG91bGQgSSBrbm93IGFib3V0IHRyYXZlbGxpbmcgd2l0aCBteSBjaGlsZCBhZnRlciB0aGVpciBraWRuZXkgdHJhbnNwbGFudD8g	Question	FAQ ABOUT TRAVELING WITH A KIDNEY TRANSPLANT!, Cindy Flores, 2019‐Nov‐19	Independent (patient)	United States	Patient (Adult)	Yes	1955	71
3	https://www.youtube.com/watch?v=BvxXdqjlH6Q&pp=ygVRV2hhdCBzaG91bGQgSSBrbm93IGFib3V0IHRyYXZlbGxpbmcgd2l0aCBteSBjaGlsZCBhZnRlciB0aGVpciBraWRuZXkgdHJhbnNwbGFudD8g	Question, keyterm	Travel After Transplantation, Leonardo Riella, 2018‐Feb‐04	Hospital	United States	Patient (Adult)	No	637	12
Mental health									
1	https://www.youtube.com/watch?v=PdX4VA2dNMs&pp=ygVQSG93IGRvIEkgdGFrZSBjYXJlIG9mIG15IGNoaWxk4oCZcyBtZW50YWwgaGVhbHRoIGFmdGVyIHRoZWlyIGtpZG5leSB0cmFuc3BsYW50PyA%3D	Question, keyterm	Solid Organ Transplant's Impact on Mental Health: Explained | Cincinnati Children's, Katherine Bedard‐Thomas and Kristin Rich, 2021‐Mar‐21	Hospital	United States	Caregiver (Pediatric)	No	394	3
2	https://www.youtube.com/watch?v=hshjH9VxyLQ&pp=ygVQSG93IGRvIEkgdGFrZSBjYXJlIG9mIG15IGNoaWxk4oCZcyBtZW50YWwgaGVhbHRoIGFmdGVyIHRoZWlyIGtpZG5leSB0cmFuc3BsYW50PyA%3D	Question	Helping Your Child Manage Their Kidney Transplant | Rare Disease | Cystinosis, American Kidney Fund, 2021‐Jul‐28	Healthcare Organization	United States	Caregiver (Pediatric)	No	1746	6
3	https://www.youtube.com/watch?v=yeO9ScNU8Pk&pp=ygVQSG93IGRvIEkgdGFrZSBjYXJlIG9mIG15IGNoaWxk4oCZcyBtZW50YWwgaGVhbHRoIGFmdGVyIHRoZWlyIGtpZG5leSB0cmFuc3BsYW50PyA%3D	Question	10 Tips And Advice | Life After A Kidney Transplant, Crissy Mac, 2021‐Feb‐07	Independent (patient)	United States	Caregivers and Adult patients	Yes	7918	315

### 3.3 Healthcare Provider Reviews

Healthcare providers conducted reviews on 125 of 128 resources since 3 links were unavailable. Each resource was reviewed by two independent reviewers, and they exhibited agreement in 93% (816/875) of the assessment parameters. The first and second reviewers agreed that they would recommend 45% of the Google and 24% of the YouTube resources to a caregiver and just 32% of the Google and 24% of the YouTube resources to a pediatric patient. Of the resources agreed to be suitable for pediatric patients, only 14% of the Google and 30% of the YouTube resources were determined to be useful for patients younger than 10. The majority were determined to be appropriate for patients > 14.

**FIGURE 2:**
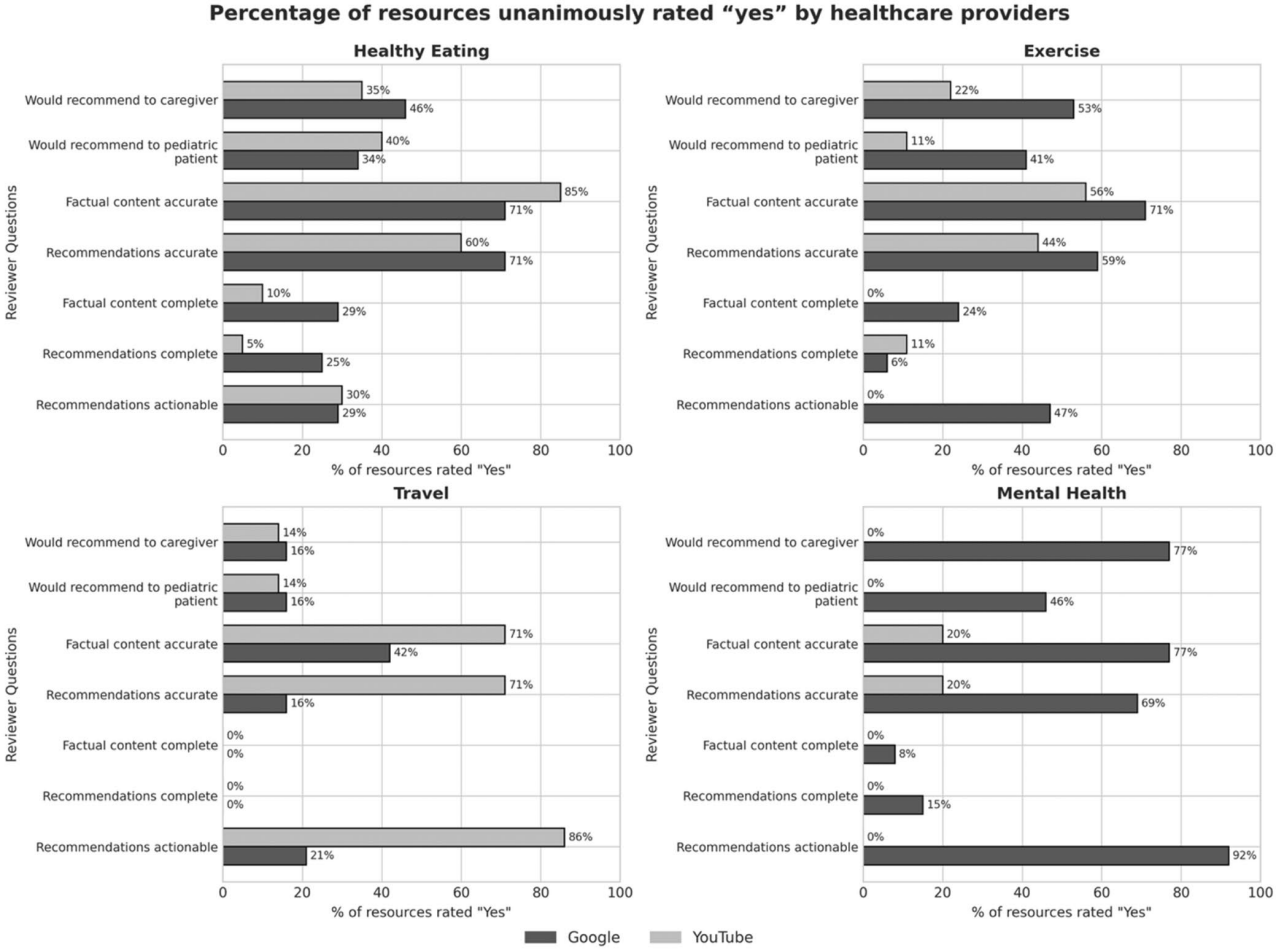
Percentage of resources unanimously rated “yes” by healthcare providers.

Both reviewers perceived the majority of resources to be accurate (65% for Google, 68% for YouTube), but only 18% of Google and 5% of YouTube resources were agreed to be factually complete. There was agreement that 12% of Google and 54% of YouTube resources contained “complete recommendations” while they considered 42% of the Google and 29% of the YouTube recommendations to be actionable. (Figure 2). Forty eight of 125 resources reviewed were agreed to be suitable for pediatric kidney recipients or caregivers. Within this group, 97% of Google and 92% of YouTube resources were considered accurate. Furthermore, 89% of Google, and 85% of YouTube recommendations were deemed accurate. Ratings were lower in the assessments of factual (Google 31%, YouTube 7%) and recommendation completeness (Google 31%, YouTube 15%). Actionable recommendations were noted in 71% Google and 54% of the YouTube resources.

Overall, many reviewers described the resources as clear, comprehensive, and practically useful, noting strong content in areas such as food safety and caregiver support. At the same time, reviewers frequently observed that most resources were oriented towards adult transplant recipients, limiting their applicability to pediatric audiences. Several comments also highlighted that many resources were centered on institution‐specific protocols, which, while useful locally, were not broadly generalizable. Reviewers additionally noted that a number of resources did not fully address the search‐term questions. For example, some materials labeled as nutrition resources focused primarily on general post‐transplant care rather than providing topic‐specific dietary guidance. Illustrative explanations for why resources did not meet specific assessment criteria (e.g., accuracy, completeness, actionability) are provided in Table 5.

**TABLE 5:** Exemplar explanations for why a resource was not rated “yes” by healthcare providers.

Reviewer questions	Explanation
3. Is the factual content accurate?	“You don't need to avoid ‘raw foods’—this would be misleading for someone who thought you should avoid raw fruit/veg”; “States that ‘you are able to travel as much as you wish after transplant’—this is misleading as travel is largely dependent on medical stability and health…”
4. Are the recommendations accurate?	“Unsure what ‘minimal sugar requirement’ would be. Unsure what ‘liberalized salt’ means.”; “social media engagement may or may not be a health coping strategy, especially for children and teens”
5. Is the factual content complete?	“doesn't provide information on how to prepare food safely”; “There is no information about long term physical activity or how to resume exercise”
6. Are the recommendations complete?	“There is no recommendation regarding fluid intake”; “A specific recommendation against motorized sports such as dirt biking and ATV riding should be included”
7. Are the recommendations actionable?	“Need specific suggestions for meal choices and portions (i.e., stating protein need as 1 g/kg/day is not patient or family friendly).”; “does not describe how to go about adjusting medication dosing times for time zone changes, or contacting transplant coordinator for guidance…simply states you should ‘think about how the time change might affect when to give medications’”

### 3.4 Readability

Based on the Average Reading Level Assessment Calculator, the readability scores for the assessed materials had a grade score range of 5 to 17. The average reading level was Grade 9 for resources on healthy eating, Grade 10 for those on exercise and travel, and Grade 11 for mental health‐related resources. Only six of the 82 resources identified via a Google search and assessed using the readability scoring tool, scored a grade of 6 or lower (Healthy Eating: *n* = 3, grade 6; Exercise: *n* = 1, grade 6; Travel: *n* = 1, grade 5; Mental Health: *n* = 1, grade 6). For resources that scored a grade of 6 or lower, the readability scores were similar for the Flesch Reading Ease (score = 66–81), Gunning Fog (score = 6.4–8.5), Flesch–Kincaid Grade Level (score = 4.17–5.18), Coleman‐Liau Index (score = 6.3–8.62), SMOG Index (score = 5.4–5.73), and Forcast Readability Formula (score = 9.43–11.43), with some variability in readability scores between Automated Readability Index (score = 3.58–6.03) and Linsear Write Formula (score = 1.99–5.38). Reviewers agreed that 28 of the Google resources were suitable for pediatric transplant recipients; the readability scores for this subset mirrored the entire group at 6–17. A full overview of the readability scores for resources identified via a Google search is available in the Supporting Information S7.

### 3.5 Understandability/Actionability

**FIGURE 3:**
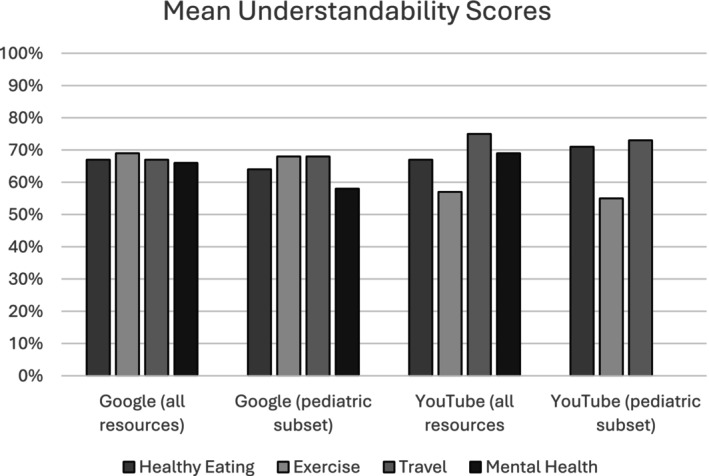
Mean understandability scores according to PEMAT across all topics.

Overall, the articles and videos included in this review performed poorly regarding understandability and actionability. According to the PEMAT developers a threshold of 70% is considered the minimum score required for a web‐based resource to be considered actionable and understandable [26]. In the present study, the mean understandability score for resources found on both Google YouTube was 67%. In the subset of resources determined to be suitable for pediatric kidney recipients, the mean understandability score was 64% for Google and 70% for YouTube. Articles found on Google infrequently defined medical terminology or used common language and inconsistently included a summary or visual materials (points deducted for items 3, 4, 11, and 15), while videos on YouTube similarly lost points for items 3, 4, and 11. Figure 3 presents the mean understandability scores according to PEMAT across all topics.

**FIGURE 4:**
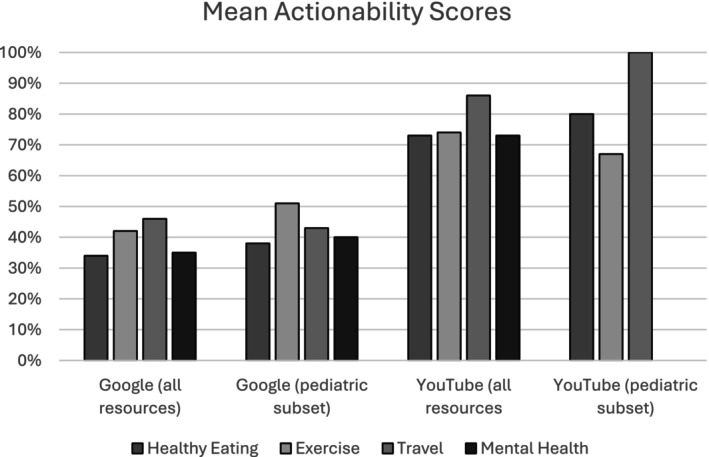
Mean actionability scores according to PEMAT across all topics.

The average actionability score for resources found on Google was 39% and YouTube was 77%. Most of the resources found on Google scored 2/5 points (40%). The average actionability score for the articles determined to be appropriate for pediatric recipients was 43% for Google and 81% for YouTube. Resources typically addressed the user directly and identified at least one action they could take (i.e., receiving points for items 20 and 21) but failed to break these actions down into manageable steps, provide meaningful tools to assist users or provide visual aids to help users act on instructions (i.e., points deducted for items 22, 23, and 26). Similarly, the average actionability score for YouTube videos was 2/3 (67%). These videos scored points for addressing users directly and identifying actions users could take (i.e., items 20 and 21) but did not break down actions into manageable steps (i.e., item 22). Figure 4 presents the mean actionability scores according to PEMAT across all topics.

The two independent reviewers (AB and RRDC) unanimously agreed in their assessment of understandability and actionability in 126/128 resources (98% agreement). The disagreements were minor and easily resolved by a third reviewer (HM). A full overview of all understandability and actionability scores is available in Supporting Information S8.

## 4 Discussion

This study assessed the availability, quality, and readability of online educational resources related to pediatric kidney transplantation. Using both patient‐centered and medically oriented search strategies on Google and YouTube, we aimed to simulate real‐world information‐seeking behaviors of patients and caregivers. Our findings highlight several key insights and gaps in the current landscape of online transplant education.

Searches using patient‐style questions consistently yielded more relevant educational resources than those using medical key terms, especially with Google. While key terms often led to academic or research‐focused content, question‐based searches were more likely to identify patient‐directed materials. Despite an extensive search strategy, the number of high‐quality, pediatric‐specific resources was lower than anticipated. Most resources were designed for adult transplant recipients, and only a minority were tailored to pediatric audiences and even fewer involved patients in their creation. This is concerning given the unique educational needs of pediatric patients and their caregivers.

PEMAT scores indicated moderate understandability (mean 67%) and wide variation in actionability. Google resources scored low on actionability (mean 39%), while YouTube performed better (mean 77%), although both frequently lacked stepwise instructions and concrete tools. Healthcare provider reviews offered important context: while many resources were factually accurate, far fewer were considered factually complete and actionable. Reviewers often withheld recommendation unless resources were not only accurate but also comprehensive and actionable within the context of the question. This suggests that factual correctness alone is insufficient; resources must also provide clear, complete, and practical guidance to be recommended by healthcare providers.

Readability posed an additional challenge. Most materials exceeded recommended reading levels, with average scores between Grade 9–11, and only six of 82 Google resources meeting the Grade 6 benchmark. This may limit comprehension for caregivers with lower health literacy and for children and adolescents navigating transplant care.

Few pediatric‐focused resources were identified across topics, which likely reflects both limited availability and poor discoverability. Our search strategy intentionally used broad, topic‐based terms to mirror typical caregiver behavior, as parents usually begin with simple condition‐related queries rather than age‐specific modifiers. Pilot testing of alternatives (e.g., “child,” “kid,” “pediatric transplant diet”) did not meaningfully increase the yield of pediatric‐specific content. Thus, the scarcity of pediatric results likely represents a true gap in accessible online resources, rather than a limitation of the search strategy alone. The lack of easily identifiable pediatric‐appropriate content highlights an important vulnerability for families seeking transplant guidance online.

Since our intent was to evaluate resources based on how well they addressed specific search parameters mimicking a real‐world search for pediatric transplant information, we assessed all resources through a pediatric lens, even if they were originally created for adult audiences. Many resources identified through our searches did not directly answer the search questions used. For example, Google searches often yielded lengthy program booklets (50–70 pages) intended to provide comprehensive overviews of the transplant process. While these documents were thorough, the information relevant to our selected topics was often buried and difficult to locate.

Only 28 of the 125 resources assessed by our reviewers were considered suitable for education of pediatric kidney transplant recipients. Within this subset, the average reading level, actionability, and understandability scores were similar to the larger group. One notable difference in pediatric appropriate resources was that no YouTube Mental Health resources were considered appropriate for pediatric patients. Additionally, many resources appeared to be tailored to the needs of specific regions or healthcare programs. For instance, the YouTube search for healthy eating returned several videos created by physicians in India, which focused on local food safety concerns; these resources contained complete and accurate information applicable to areas where food and water safety is of key concern. We also identified patient‐created videos, often part of a “transplant journey” series. These were engaging and valuable in capturing lived experiences but were not designed to provide comprehensive educational content. For example, one resource identified through the mental health search, *Patient Stories: Conversations with Claire* (Children's Wisconsin), was rated as incomplete in terms of factual content, recommendations, and actionability, yet was still recommended by reviewers for both caregivers and patients. This suggests that while standardized tools like PEMAT and our reviewer checklist are essential for evaluating educational quality, they may not fully capture other meaningful attributes—such as emotional resonance, relatability, or the value of lived experience. These findings highlight the importance of using multiple complementary assessment approaches to ensure a more holistic evaluation of educational resources.

Importantly, our evaluation focused on how well each resource addressed the search parameters we defined, rather than whether the resource fulfilled the creator's intended purpose or target audience. In addition to identifying resources that lacked direct relevance, some resources that pertained to our search were not identified. For example, one reviewer noted that a reputable resource on exercise did not appear in our Google search. This highlights the limitations patients can encounter with online information seeking and indicates a need for healthcare providers to play a more active role in guiding patients and caregivers to high‐quality, vetted online resources. Moreover, only a small fraction of resources showed evidence of patient involvement, representing a missed opportunity for meaningful engagement. Co‐creating educational materials with patients and caregivers may enhance relevance, cultural sensitivity, and usability.

This study has several limitations. First, there is no validated tool for assessing the accuracy of pediatric kidney transplant education materials, so we developed a custom review form for this purpose. While our healthcare provider review panel had expertise in pediatric transplant nephrology, we lacked a transplant dietitian and psychologist, which would have been valuable for assessing resources specific to healthy eating and mental health. Due to the dynamic nature of online content, some resources became unavailable during the review process. While we attempted to simulate real‐world searches, results may vary based on user location, search history, and platform algorithms. Our study was conducted prior to the artificial intelligence feature that has been since incorporated into Google searches. Future study is recommended to assess the accuracy of these summaries.

## 5 Conclusion

The use of Google and YouTube to find information is where people turn to find quick answers, and this is no different for patients and caregivers. Our review illustrates that while accurate and actionable resources can be found via Google and YouTube, there is a significant risk of locating resources that do not address specific search parameters. Our findings suggest that healthcare providers should play a more active role in guiding patients and caregivers to high‐quality, vetted online resources. Given the variability in quality and the potential for misinformation, curated lists of recommended websites or videos could help bridge the gap between clinical education and independent learning.

## Funding

The project was funded by the Kidney Foundation of Canada (Grant 2023AHKRG‐1144136).

## Conflicts of Interest

The authors declare no conflicts of interest.

## Supporting information


**Appendix S1:** CST pediatric education initiative list of education topics.

**Supporting Information S1:** Readability formulas used to assess resources.
**Supporting Information S2:** PEMAT‐P for assessing understandability and actionability.
**Supporting Information S3:** PEMAT‐AV for assessing understandability and actionability.
**Supporting Information S4:** Identification of resources through Google and YouTube Search.
**Supporting Information S5:** Google search results by topic.
**Supporting Information S6** YouTube search results by topic.
**Supporting Information S7:** Readability scores for objective 1 (Google search).
**Supporting Information S8:** PEMAT‐P and PEMAT‐AV understandability and actionability scores.

## Data Availability

Data sharing not applicable to this article as no datasets were generated or analysed during the current study.
