# Supplementary material for: How Well Do “Dr. Google” and “Professor YouTube” Answer Health Questions for Pediatric Kidney Transplant Recipients and Caregivers?
Source: Pediatr Transplant. 2026 Aug 11;30(8):e70409. doi: 10.1111/petr.70409 (PMC13458959; doi:10.1111/petr.70409)
Supplement: Supplementary file 1 [file PETR-30-e70409-s001.docx]

**Supplement 1. Readability formulas used to assess resources**

| Readability Formulas | Description | Interpretation |
| --- | --- | --- |
| Flesch Reading Ease Formula^20^ | Scores text on average syllables per word and average words per sentence on a scale of 0 to 100, with higher scores suggesting easier reading. | 90-100: very easy  80-89: easy  70-79: fairly easy  60-69: standard  50-59: fairly difficult  30-49: difficult  0-29: very confusing |
| Gunning Fog Index^21^ | Scores reading levels from 6 to 17 by averaging sentence length and calculating the proportion of complex words (defined as words with 3+ syllables). | Score corresponds to a specific United States grade level. Any score above 12 is regarded as post-secondary level. |
| Automated Readability Index^22^ | Analyzes characters in each word and words in each sentence to estimate the reading difficulty and correlate it to the grade level required for text comprehension. |  |
| Flesch-Kincaid Grade Level^23^ | Considers the sentence length and the complexity of words to measure the reading difficulty and assist in matching readers’ skills to appropriate material. |  |
| Coleman-Liau Index^24^ | Scores readability based on average number of characters per word and average sentence length. |  |
| SMOG Index^25^ | Used to evaluate reading difficulty, specifically in texts containing a minimum of 30 sentences. Uses polysyllabic terms to determine the grade level. |  |
| Linsear Write Readability Formula^26^ | Focuses on number of words with a minimum of 3 syllables and sentence length to determine the complexity of the text. |  |
| Forecast Readability Formula^27^ | Derived on the number of 1 syllable words in a 150-word passage and does not factor in sentence length. Designed for non-narrative texts, such as forms and multiple-choice exams. |  |
| Average Reading Level Consensus Calculator^19^ | Uses 8 widely used readability algorithms (listed above) to produce an estimated reading difficulty score and grade level. |  |
|  |  |  |

**Supplement 2. PEMAT-P for assessing Understandability and Actionability**

| **Understandability Domain** | |
| --- | --- |
| Topic | Item number and description |
| Content | 1. The material makes its purpose completely evident. |
|  | 2. The material does not include information or content that distracts from its purpose. |
| Word Choice & Style | 3. The material uses common, everyday language. |
|  | 4. Medical terms are used only to familiarize audience with the terms. When used, medical terms are defined. |
|  | 5. The material uses the active voice. |
| Use of numbers | 6. Numbers appearing in the material are clear and easy to understand. |
|  | 7. The material does not expect the user to perform calculations. |
| Organization | 8. The material breaks or "chunks" information into short sections. |
|  | 9. The material's sections have informative headers. |
|  | 10. The material presents information in a logical sequence. |
|  | 11. The material provides a summary. |
| Layout & Design | 12. The material uses visual cues (e.g., arrows, boxes, bullets, bold, larger font, highlighting) to draw attention to key points. |
| Use of Visual Aids | 15. The material uses visual aids whenever they could make content more easily understood (e.g., illustration of healthy portion size). |
|  | 16. The material’s visual aids reinforce rather than distract from the content. |
|  | 17. The material’s visual aids have clear titles or captions. |
|  | 18. The material uses illustrations and photographs that are clear and uncluttered. |
|  | 19. The material uses simple tables with short and clear row and column headings. |
| **Actionability Domain** | |
| 20. The material clearly identifies at least one action the user can take. | |
| 21. The material addresses the user directly when describing actions. | |
| 22. The material breaks down any action into manageable, explicit steps. | |
| 23. The material provides a tangible tool (e.g., menu planners, checklists) whenever it could help the user take action. | |
| 24. The material provides simple instructions or examples of how to perform calculations. | |
| 25. The material explains how to use the charts, graphs, tables, or diagrams to take actions. | |
| 26. The material uses visual aids whenever they could make it easier to act on the instructions. | |

Source: PEMAT-P Tool

**Supplement 3. PEMAT-AV for assessing Understandability and Actionability**

| **Understandability Domain** | |
| --- | --- |
| Topic | Item number and description |
| Content | 1. The material makes its purpose completely evident. |
| Word Choice & Style | 3. The material uses common, everyday language. |
|  | 4. Medical terms are used only to familiarize audience with the terms. When used, medical terms are defined. |
|  | 5. The material uses the active voice. |
| Organization | 8. The material breaks or "chunks" information into short sections. |
|  | 9. The material's sections have informative headers. |
|  | 10. The material presents information in a logical sequence. |
|  | 11. The material provides a summary. |
| Layout & Design | 12. The material uses visual cues (e.g., arrows, boxes, bullets, bold, larger font, highlighting) to draw attention to key points. |
| Use of Visual Aids | 13. Text on the screen is easy to read. |
|  | 14. The material allows the user to hear the words clearly (e.g., not too fast, not garbled). |
|  | 18. The material uses illustrations and photographs that are clear and uncluttered. |
|  | 19. The material uses simple tables with short and clear row and column headings. |
| **Actionability Domain** | |
| 20. The material clearly identifies at least one action the user can take. | |
| 21. The material addresses the user directly when describing actions. | |
| 22. The material breaks down any action into manageable, explicit steps. | |
| 25. The material explains how to use the charts, graphs, tables, or diagrams to take actions. | |

Source: PEMAT-AV Tool

**Supplement 4. Identification of resources through Google and YouTube Search**

**Supplement 5. Google Search Results by topic**

| # | URL/Reference | Search Phrase | Title, Authors, Date | Format of Resource | Type of Organization Producing Resource | Country of Origin | Target audience | Patient Involvement |
| --- | --- | --- | --- | --- | --- | --- | --- | --- |
| Healthy eating | | | | | | | | |
| 1 | [https://www.ucsfbenioffchildrens.org/education/nutrition-after-kidney-transplant#:~:text=Choose%20low%2Dfat%20and%20low,activities%20are%20allowed%20after%20transplant.](https://www.ucsfbenioffchildrens.org/education/nutrition-after-kidney-transplant#:~:text=Choose%20low%2Dfat%20and%20low,activities%20are%20allowed%20after%20transplant) | question, keyterm | Nutrition After Kidney Transplant, UCSF Benioff Children's Hospital medical specialists, unknown | Website | Hospital | USA | Caregivers (of Pediatric patients) | No |
| 2 | <https://www.kidney.org/atoz/content/foods-avoid-after-transplantation> | question | Foods to Avoid After Transplantation, National Kidney Foundation, 04/19 | Website | Healthcare Organization | USA | Patients (Adult) | No |
| 3 | <https://www.med.umich.edu/mott/pdf/transplant/pediatric-kidney-transplant-diet-nurtrition.pdf> | question, keyterm | Kidney Transplant Nutrition Therapy, C.S Mott Children's Hospital, unknown | Online booklet | Hospital | USA | Caregivers  (of Pediatric patients) | No |
| 4 | <https://columbiasurgery.org/kidney-transplant/nutrition-after-renal-transplant> | question, keyterm | Nutrition After Renal Transplant, Columbia University, unknown | Website | Hospital | USA | Patients  (Adult) | No |
| 5 | <https://myhealth.alberta.ca/KidneyTransplant/transplant-recipient-information/nutrition-before-and-after-your-transplant/nutrition-after-your-transplant> | question, keyterm | Nutrition after your transplant, Kidney Health Section, Medicine Strategic Clinical Network, Alberta Health Services, 09/26/2022 | Website | Government (Health Region) | Canada | Patients  (Adult) | No |
| 6 | <https://www.choa.org/-/media/Files/Childrens/teaching-sheets/nutrition-and-kidney-transplant.pdf> | question | Nutrition and Kidney Transplant, Children's Healthcare of Atlanta, 02/2022 | Online booklet | Hospital | USA | Caregivers (of Pediatric patients) | No |
| 7 | <https://teens.aboutkidshealth.ca/Article?contentid=2698&language=English> | question | Monitoring your diet and fluid intake after a kidney transplant, SickKids Staff, 11/30/2017 | Website | Hospital | Canada | Patients (pediatric) | No |
| 8 | <https://www.youtube.com/watch?time_continue=1&v=04JtzMiZsQk&embeds_referring_euri=https%3A%2F%2Fteens.aboutkidshealth.ca%2F&source_ve_path=Mjg2NjY&feature=emb_logo> | question | Transplant - Session 8: Diet, Teens Taking Charge/ SickKids Hospital, 4/3/2018 | YouTube Video | Hospital | Canada | Patients (pediatric) | Yes |
| 9 | <https://www.infokid.org.uk/supporting-information/healthy-eating-after-kidney-transplant/> | question, keyterm | Healthy eating after a kidney transplant, he British Association of Paediatric Nephrology (BAPN) and Kidney Care UK, unknown | Website | Healthcare Organization | UK | Caregivers (of Pediatric patients) | No |
| 10 | <https://www.clevelandclinicabudhabi.ae/en/health-hub/health-resource/treatments-and-procedures/healthy-eating-after-your-kidney-transplant> | question | Healthy Eating After Your Kidney Transplant, Cleveland Clinic Abu Dhabi, 2017 | Website | Hospital | USA/United Arab Emirates | Patients (Adult) | No |
| 11 | <https://www.albertahealthservices.ca/assets/info/nutrition/if-nfs-eating-well-after-kidney-transplant.pdf> | question | Eating well after a kidney transplant, Alberta Health Services: Nutrition Services, 02/2023 | Online booklet | Government (Health Region) | Canada | Patients (Adult) | No |
| 12 | <https://www.umc.edu/Healthcare/Transplant/Nutrition-After-Transplant.html> | question, keyterm | Nutrition After Transplant, UMMC Health care, unknown | Website | Hospital (Group) | USA | Patients (Adult) | No |
| 13 | <https://www.mayoclinic.org/tests-procedures/kidney-transplant/about/pac-20384777> | question, keyterm | Kidney transplant: Overview, Mayo Clinic Staff, 01/19/2024 (last update) | Website | Hospital | USA | Patients (Adult) | No |
| 14 | <https://patient.uwhealth.org/healthfacts/494> | question | Nutrition Guidelines After Solid Organ Transplant, Nutrition clinics for UW Hospital and Clinics (UWHC) - Department of Nursing, 07/2020 | Website | Hospital (Group) | USA | Patients (Adult) | No |
| 15 | <https://nutrikonnect.in/kidney-transplant-diet-dos-and-donts-to-keep-in-mind-post-kidney-transplant-2/> | question | Kidney transplant diet: do’s and dont’s to keep in mind post-kidney transplant, Dr. Rachana Jasani PhD RD, unknoqn | Website | Independent users | India | Patients (Adult) | No |
| 16 | <https://www.emeesykidney.nhs.uk/images/Users/Dietetic_resources/Healthy_Eating_After_Your_Transplant_Paeds.pdf> | question | Healthy Eating After Your Kidney Transplant: Information for children with kidney disease, Nottingham Children's Hospital: Department of Dietetics and Nutrition, 1/2016 | Online booklet | Hospital | UK | Patients (pediatric) | No |
| 17 | <https://health.ucdavis.edu/transplant/PDFs/posttransplant/Nutrition%20After%20A%20Kidney%20Transplant.pdf> | question | Nutrition after a Kidney Transplant, Clinical Dietitians, Food & Nutrition Services, UC Davis Medical Center, 07/2018 | Online booklet | Hospital (Group) | USA | Patients (Adult) | No |
| 18 | <https://www.uchicagomedicine.org/forefront/transplant-articles/safe-preparation-and-foods-to-avoid-after-an-organ-transplant> | question | Food safety and foods to avoid after organ transplant, Jamie Bartosch, 1/16/2020 | Website | Hospital | USA | Patients (Adult) | No |
| 19 | <https://www.cuh.nhs.uk/patient-information/dietary-advice-following-a-kidney-kidney-pancreas-transplant/> | question | Dietary advice following a kidney/kidney-pancreas transplant, Nutrition and dietetics department Cambridge University Hospitals, 1/29/2020 | Website | Hospital | England | Patients (Adult) | No |
| 20 | <https://www.gleneagles.com.sg/health-plus/article/kidney-transplant-dietary-advice> | question | Life After a Kidney Transplant - Dietary Advice to Know, Gleneagles Hospital, 2/25/2021 | Website | Hospital | Singapore | Patients (Adult) | No |
| 21 | <https://www.myast.org/sites/default/files/Pediatric%20Kidney%20Transplantation%20BrochureAST%20%20-%20final%20copy%202015-06-27_0.pdf>  <https://www.myast.org/uploads/files/general/Pediatric-Kidney-Transplantation-1-1.pdf>. | question | Pediatric Kidney Transplantation: Guide for Patients and Families, American Society of Transplantation, 06/2015 | Online booklet | Healthcare Organization | USA | Caregivers (of Pediatric patients) | No |
| 22 | <https://www.kuh.ku.edu.tr/our-centers/organ-transplant-center/kidney-transplant/kidney-transplant-n-children> | question | Kidney Transplant in Children, KOC University Hospital, unknown | Website | Hospital | Istanbul | Caregivers (of Pediatric patients) | No |
| 23 | <https://nephcure.org/livingwithkidneydisease/kidney-failure/transplant/> | question | Kidney Transplants, Nephcure, unknown | Website | Healthcare Organization | USA | Patients (Adult) | No |
| 24 | <https://transplant.jacksonhealth.org/programs/kidney-transplant/> | question | Kidney Transplant Overview, UHealth Jackson: Miami Transplant Institute, unknown | Website | Hospital | USA | Patients (Adult) | No |
| 25 | <https://www.lhsc.on.ca/media/9395/download> | question | Kidney Transplantation: Patient Handbook After Your Kidney Transplant, London Health Sciences Centre: Multi-Organ Transplant Program, 7/15/2022 | Online booklet | Hospital (Group) | Canada | Patients (Adult) | No |
| 26 | <https://www.nebraskamed.com/sites/default/files/documents/Transplant/pancreas%20education/nutrition.pdf> | question | Your Guide to Healthy Eating After Your Kidney and/or Pancreas Transplant, Nebraska Medicine, unknown | Online booklet | Hospital | USA | Patients (Adult) | No |
| 27 | <https://renal.providencehealthcare.org/sites/renal.providencehealthcare.org/files/Kidney-Transplant-Recipient-Booklet.pdf> | question | Living with Kidney Transplantation, Providence Health Care, 03/2011 | Online booklet | Government (Health Region) | Canada | Patients (Adult) | No |
| 28 | <https://www.kidney.org/atoz/content/nutrichild> | keyterm | Nutrition for Children with Chronic Kidney Disease, Council on Renal Nutrition: National Kidney Foundation, 04/2019 | Website | Healthcare Organization | USA | Caregivers (of Pediatric patients) | No |
| 29 | <https://www.myast.org/uploads/files/general/DASH-Diet-for-Pediatric-Kidney-Transplant-Recipients_Final.pdf> | keyterm | DASH Diet for Pediatric Kidney Transplant Recipients, Kristen Sgambat PhD RD (American Society of Transplantation), 2018 | Online booklet | Healthcare Organization | USA | Caregivers and Pediatric patients | No |
| 30 | <https://www.transplantmanitoba.ca/uploads/ck/files/12%20Pre%20Transplant%20Interim%20Ed_%20Healthy%20Eating.pdf> | keyterm | Pre-Transplant Education: Healthy Eating, Children's Hospital Foundation of Manitoba/Children's Hospital: Health Sciences Centre Winnipeg, unknown | Online booklet | Hospital | Canada | Patients (pediatric) | No |
| 31 | <https://www.transplantmanitoba.ca/uploads/ck/files/healthy%20living%20to%20avoid%20infections%202015-07-15.pdf> | keyterm | Healthy Living with a Transplant to Avoid Infections, Transplant Manitoba: Pediatric Kidney Program, unknown | Online booklet | Healthcare Organization | Canada | Patients (pediatric) | No |
| 32 | <https://www.davita.com/diet-nutrition/articles/basics/the-transplant-diet> | keyterm | The Transplant Diet, Davita Kidney Care, unknown | Website | Healthcare Organization | USA | Patients (pediatric) | No |
| 33 | <https://www.nationwidechildrens.org/specialties/kidney-transplant-program/kidney-transplant-health-tips-for-teens> | keyterm | Kidney Transplant Health Tips for Teens, Nationwide Children's Hospital, unknown | Website | Hospital | USA | Patients (Adult) | No |
| 34 | <https://www.rileychildrens.org/health-info/kidney-transplant> | keyterm | Kidney Transplant, Riley Children's Health: Indiana University Health, unknown | Website | Hospital | USA | Caregivers (of Pediatric patients) | No |
| 35 | <https://flipbook.childrensmercy.org/kidney-transplant-education-book/download-pdf.html> | keyterm | Pediatric Kidney Transplant Education Book: A Guide for Patients and Families, The Kidney Transplant Team at Children's Mercy | Online booklet | Hospital | USA | Caregivers and Pediatric patients | Yes |
| Exercise | | | | | | | | |
| 1 | [https://www.aboutkidshealth.ca/Article?contentid=3952&language=English&hub=transplant#:~:text=Your%20child%20should%20avoid%20heavy,house%20or%20playing%20active%20games.](https://www.aboutkidshealth.ca/Article?contentid=3952&language=English&hub=transplant#:~:text=Your%20child%20should%20avoid%20heavy,house%20or%20playing%20active%20games) | question | Physical activity and sport post kidney transplant, Sickkids Staff (SickKids Hospital), 5/12/2021 | Website | Hospital | Canada | Caregivers (Pediatric) | No |
| 2 | <https://teens.aboutkidshealth.ca/Article?contentid=2699&language=English> | question, keyterm | Physical activity after a kidney transplant, SickKids Staff (SickKids Hospital), 11/30/2017 | Website | Hospital | Canada | Patients (Pediatric) | No |
| 3 | <https://www.youtube.com/watch?v=FL_E4vvRP0M&t=2s> | question | Transplant - Session 9: Lifestyle and Exercise, AboutKidsHealth - The Hospital for Sick Children, 4/03/2018 | YouTube Video | Hospital | Canada | Patients (Pediatric) | Yes |
| 4 | <https://health.clevelandclinic.org/kidney-transplant-recovery/> | question, keyterm | 11 Kidney Transplant Tips, Cleveland Clinic, 09/28/2022 | Website | Hospital | USA | Patients (Adult) | No |
| 5 | <https://www.imperial.nhs.uk/our-services/kidney-and-transplant/kidney-and-pancreas-transplantation/kidney-transplantation/living-with-a-transplant> | question | Living with Kidney Transplant, NHS, unknown | Website | Government | UK | Patients (Adult) | No |
| 6 | <https://www.chp.edu/our-services/transplant/liver/recovery/life-after/physical-therapy> | question | Physical Therapy Post-Liver Transplant, UPMC Children's Hospital of Pittsburgh | Website | Hospital | USA | Caregivers (Pediatric) | No |
| 7 | <https://www.childrensdmc.org/services/transplant-center/transplant-resources-and-links-tips-to-help-your-child-adjust-after-an-organ-transplant> | question | Tips to Help Your Child Adjust After an Organ Transplant, United Network for Organ Sharing/Children's Hospital of Michigan, unknown | Website | Hospital | USA | Caregivers (Pediatric) | N |
| 8 | <https://my.clevelandclinic.org/health/treatments/22537-kidney-transplant> | question | Kidney Transplant, Cleveland Clinic, 3/14/2022 | Website | Hospital | USA | Patients (Adult) | No |
| 9 | <https://myhealth.alberta.ca/Health/aftercareinformation/pages/conditions.aspx?hwid=ud1689> | question, keyterm | Kidney Transplant: What to Expect at Home, Healthwise Staff, 03/01/2023 | Website | Government (Health Region) | Canada | Patients (Adult) | No |
| 10 | <https://dam.upmc.com/-/media/upmc/services/transplant/documents/afterkidneypancreastransplant.pdf?la=en&rev=eaa52fb975544c2da86dd532f3b63900&hash=5D5B6C65F3377F6F6DE974EA25EC75A3> | question | What to Expect after Kidney/Pancreas Transplant, UPMC, 2020 | Online booklet | Hospital | USA | Patients (Adult) | No |
| 11 | <https://www.texaschildrens.org/sites/default/files/TX_Childrens_Kidney_Transplant_Handbook.pdf> | question | Kidney Transplant Education Book, Transplant Services: Texas Children's Hospital, unknown | Online booklet | Hospital | USA | Caregivers and Pediatric patients | No |
| 12 | <https://www.uhcw.nhs.uk/download/clientfiles/files/Patient%20Information%20Leaflets/Surgical%20Services/Renal%20Transplantation/Information%20for%20patients%20following%20renal%20transplant.pdf> | question | Information for patients following a renal transplant, University Hospitals Coventry and Warwickshire/NHS Trust, 7/2023 | Online booklet | Hospital | UK | Patients (Adult) | No |
| 13 | <https://transplanttalk.childrenshospital.org/tips-tricks/spring-2015-sports-transplant/> | keyterm | Spring 2015: Sports After Transplant, TransplantTalk: Boston Children's Hospital Pediatric Transplant Centre, 2015 | Website | Hospital | USA | Caregivers (Pediatric) | No |
| 14 | <https://www.myast.org/sites/default/files/Pediatric%20Kidney%20Transplantation%20BrochureAST%20%20-%20final%20copy%202015-06-27_0.pdf> | keyterm | Pediatric Kidney Transplantation: A Guide for Patients and Families, American Society of Transplantation, 6/2015 | Online booklet | Healthcare Organization | USA | Caregivers (Pediatric) | No |
| 15 | <https://www.mayoclinic.org/tests-procedures/kidney-transplant/about/pac-20384777> | keyterm | Kidney Transplant, Mayo Clinic Staff, 1/19/2024 | Website | Hospital | USA | Patients (Adult) | No |
| 16 | <https://columbiasurgery.org/kidney-transplant/resuming-life-after-kidney-transplantation> | keyterm | Resuming Life After Kidney Transplant, Columbia University Irving Medical Centre, unknown | Website | Hospital | USA | Patients (Adult) | No |
| 17 | <https://www.massgeneral.org/assets/mgh/pdf/transplant/transplant-after-care.pdf> | keyterm | Caring for yourself and your transplant, Massachusetts General Hospital Transplant Team, 4/2023 | Online booklet | Hospital | USA | Patients (Adult) | Yes |
| 18 | <https://www.astellascares.com/patient/exercise-after-transplant> | keyterm | Exercise After a Transplant, Astellas Cares, unknown | Website | Industry | USA | Patients (Adult) | No |
| Travel | | | | | | | | |
| 1 | <https://www.kidney.org/atoz/content/traveltip> | question | Travel Tips: A Guide for Kidney Patients, National Kidney Foundation, unknown | Website | Healthcare Organization | USA | Patients (Adult) | No |
| 2 | <https://columbiasurgery.org/kidney-transplant/resuming-life-after-kidney-transplantation> | question | Resuming Life After Kidney Transplantation, Columbia University Irving Medical Centre, unknown | Website | Hospital | USA | Patients (Adult) | No |
| 3 | <https://www.kidneyfund.org/kidney-donation-and-transplant/life-after-transplant-rejection-prevention-and-healthy-tips/recovery-after-transplant-surgery> | question | Recovery after transplant surgery, American Kidney Fund's Medical Advisory Committee, 02/16/2022 | Website | Healthcare Organization | USA | Patients (Adult) | No |
| 4 | <https://www.lhsc.on.ca/media/9395/download> | question | Kidney Transplantation: Patient Handbook After Your Kidney Transplant, London Health Sciences Centre: Multi Organ Transplant Program, 07/15/2022 | Online booklet | Hospital | Canada | Patients (Adult) | No |
| 5 | <https://optn.transplant.hrsa.gov/media/2464/weparentntk_optn.pdf> | question | What Every Parent Needs to Know: A guide when your child needs a transplant, U.S Organ Procurement and Transplant Network Patient Affairs Committte, and United Network for Organ Sharing staff, 2018 | Online booklet | Government | USA | Caregivers (Pediatric) | Yes |
| 6 | <https://www.chp.edu/our-services/transplant/liver/patient-family-resources/liver-transplant-safety/travel-tips> | question | Travel Tips for Pediatric Liver Transplant Patients, UPMC Children's Hospital of Pittsburgh, unknown | Website | Hospital | USA | Caregivers (Pediatric) | No |
| 7 | <https://www.myast.org/sites/default/files/Pediatric%20Kidney%20Transplantation%20BrochureAST%20%20-%20final%20copy%202015-06-27_0.pdf> | question, keyterm | Pediatric Kidney Transplantation: A Guide for Patients and Families, American Society of Transplantation, 6/2015 | Online booklet | Healthcare Organization | USA | Caregivers and Pediatric patients | No |
| 8 | <https://www.uhn.ca/Transplant/Kidney_Transplant_Program/Kidney_Transplant/Documents/kidney_transplant_manual.pdf> | question | Kidney Transplant Guide, Multi-Organ Transplant Program: Toronto General Hospital, 6/2015 | Online booklet | Hospital | Canada | Patients (Adult) | No |
| 9 | <https://myhealth.alberta.ca/KidneyTransplant/transplant-recipient-information/pregnancy-after-transplant> | question | Travel and pregnancy after transplant, Kidney Health Section: Alberta Health Services, 9/26/2022 | Website | Government | Canada | Patients (Adult) | No |
| 10 | <https://www.hopkinsmedicine.org/health/conditions-and-diseases/coronavirus/traveling-after-an-organ-transplant-during-the-covid19-era> | question | Traveling After an Organ Transplant During the COVID-19 Era, Johns Hopkins Medicine, 2/02/2021 | Website | Hospital | USA | Patients (Adult) | No |
| 11 | <https://www.nhsbt.nhs.uk/organ-transplantation/kidney/living-with-a-kidney-transplant/getting-back-to-activities/> | question | Getting back to activities after a transplant, NHS Blood and Transplant, unknown | Website | Healthcare Organization | UK | Patients (Adult) | No |
| 12 | <https://www.betterhealth.vic.gov.au/health/conditionsandtreatments/living-with-kidney-failure> | question | Living with kidney failure, Better Health Channel / Victoria State Government, 10/17/2015 | Website | Government | Australia | Patients (Adult) | No |
| 13 | <https://www.upmc.com/services/transplant/kidney-pancreas/process/faq> | question | Kidney Transplant Surgery Frequently Asked Questions, UPMC Children's Hospital of Pittsburgh, unknown | Website | Hospital | USA | Patients (Adult) | Yes |
| 14 | <https://www.imperial.nhs.uk/our-services/kidney-and-transplant/kidney-and-pancreas-transplantation/kidney-transplantation/living-with-a-transplant/travel> | question | Travel, Immerial College Healthcare / NHS Trust, 11/21/2023 | Website | Hospital | UK | Patients (Adult) | No |
| 15 | <https://www.stjoes.ca/patients-visitors/patient-education/patient-education:-k-o/after-your-kidney-transplant.pdf> | question | After Your Kidney Transplant, Renal Transplant Team St. Joseph's Healthcare, 5/2015 | Online booklet | Hospital | Canada | Patients (Adult) | No |
| 16 | <https://www.kidneyresearchuk.org/transplantation/> | question | What is Kidney Transplantation?, Kidney Research UK, unknown | Website | Healthcare Organization | UK | Patients (Adult) | No |
| 17 | <https://www.clevelandclinicabudhabi.ae/en/health-hub/health-resource/treatments-and-procedures/kidney-transplant-surgery-recovery-returning-to-normal-activities> | question | Kidney Transplant Surgery Recovery: Returning to Normal Activities, Cleveland Clinic Abu Dhabi, unknown | Website | Hospital | USA/United Arab Emirates | Patients (Adult) | No |
| 18 | <https://www.texaschildrens.org/sites/default/files/TX_Childrens_Kidney_Transplant_Handbook.pdf> | keyterm | Kidney Transplant: Transplant Education Book, Transplant Services: Texas Children's Hospital, unknown | Online booklet | Hospital | USA | Caregivers and pediatric patients | No |
| 19 | <https://www.kidney.org.uk/holiday-guidelines-for-transplant-patients> | keyterm | Holiday guidelines for transplant patients, The National Kidney Foundation, unknown | Website | Healthcare Organization | UK | Patients (Adult) | No |
| 20 | <https://www.youtube.com/watch?v=CWsEL2PTswo> | keyterm | Safe Living After Transplant -Travel Safety, American Society of Transplantation, 5/04/2022 | YouTube Video | Healthcare Organization | USA | Patients (Adult) | No |
| Mental Health | | | | | | | | |
| 1 | <https://www.kidneyfund.org/kidney-donation-and-transplant/life-after-transplant-rejection-prevention-and-healthy-tips/mental-health-and-support-after-transplant> | question, keyterm | Mental health and support after transplant, American Kidney Fund's Medical Advisory Committe, 2/16/2022 | Website | Healthcare Organization | USA | Patient (Adult) | No |
| 2 | <https://www.kidney.org/atoz/content/immunosuppression> | question, keyterm | Care after kidney transplant, National Kidney Foundation, unknown | Website | Healthcare Organization | USA | Patient (Adult) | No |
| 3 | <https://kidshealth.org/en/parents/kidney-transplant.html> | question | Kidney Transplant (for Parents), Kidney Transplant Program at Nemours Children's Health, 11/2022 | Website | Hospital (Group) | USA | Caregiver (Pediatric) | No |
| 4 | <https://transplantliving.org/community/support-groups/> | question | Support groups, UNOS Transplant Living, 4/6/2023 | Website | Healthcare Organization | USA | Caregivers and pediatric patients | No |
| 5 | <https://www.mayoclinic.org/tests-procedures/kidney-transplant/about/pac-20384777> | question, keyterm | Kidney Transplant, Mayo Clinic Staff, 1/19/2024 | Website | Hospital | USA | Patient (Adult) | No |
| 6 | <https://www.webmd.com/a-to-z-guides/life-after-transplant-tips-parents-young-children> | question | Tips for Parents of Children With Organ Transplants, WebMD Editorial Contributors, 7/19/2021 | Website | Healthcare Organization | USA | Caregiver (Pediatric) | No |
| 7 | <https://www.dpcedcenter.org/news-events/news/supporting-your-health-and-wellbeing-before-during-and-after-kidney-transplant/> | question, keyterm | Supporting Your Health and Wellbeing Before, During, and After Kidney Transplant, Elizabeth Steinberg Christofferson PhD and Leah Grande MA, 9/3/2020 | Website | Healthcare Organization | USA | Patient (Adult) | No |
| 8 | <https://www.lhsc.on.ca/media/9395/download> | question | Kidney Transplantation: Patient Handbook After Your Kidney Transplant, London Health Sciences Centre: Multi Organ Transplant Program, 07/15/2022 | Online booklet | Hospital | Canada | Patient (Adult) | No |
| 9 | <https://www.youtube.com/watch?time_continue=1965&v=e99gIdinZgE&embeds_referring_euri=https%3A%2F%2Fwww.uncmedicalcenter.org%2F&embeds_referring_origin=https%3A%2F%2Fwww.uncmedicalcenter.org&feature=emb_logo> | question | After Your Transplant -Information on Caring for Your New Organ, UNC Centre for Transplant Care, 1/6/2023 | YouTube Video | Hospital | USA | Caregivers and Adult patients | No |
| 10 | <https://stanfordhealthcare.org/medical-clinics/transplant-program/mental-health-services.html> | question, keyterm | Mental Health Services for Transplant Patients, Stanford Medicine, unknown | Website | Hospital | USA | Patients (Adult) | No |
| 11 | <https://www.massgeneral.org/assets/mgh/pdf/transplant/transplant-after-care.pdf> | question | Caring for yourself and your transplant, Massachusetts General Hospital Transplant Team, 4/2023 | Online booklet | Hospital | USA | Patients (Adult) | Yes |
| 12 | <https://www.choa.org/medical-services/transplants/teens> | keyterm | Teen Transplant Program: More Information for Parents and Caregivers, Children's Healthcare of Atlanta, unknown | Website | Hospital | USA | Caregiver (Pediatric) | No |
| 13 | <https://www.choa.org/medical-services/transplants/kidney-transplant> | keyterm | Kidney Transplant Program: What Should I Expect During My Child's Kidney Transplant Process , Children's Healthcare of Atlanta, unknown | Website | Hospital | USA | Caregiver (Pediatric) | No |
| 14 | <https://childrenswi.org/medical-care/transplant-programs/overview/kidney-transplant/patient-stories> | keyterm | Patient Stories: Conversations with Claire, Children's Wisconsin, unknown | Videos | Hospital | USA | Caregivers and Pediatric patients | Yes |

**Supplement 6. YouTube search results by topic**

| # | URL/Reference | Search Phrase | Title, Authors, Date | Type of Organization Producing Resource | Country of Origin | Target audience | Evidence of Patient Involvement | Number of video views | Number of likes |
| --- | --- | --- | --- | --- | --- | --- | --- | --- | --- |
| Healthy Eating | | | | | | | | | |
| 1 | <https://youtu.be/PKUXMOM6ZrE?si=-Z57y2PKEYv_-wSy> | Question, keyterm | Nutrition After Kidney Transplant, UCSF Benioff Children's Hospital medical specialists, unknown | Independent (Healthcare Provider) | USA | Patient (Adult) | No | 25,017 | 667 |
| 2 | <https://youtu.be/ShQNrFfEYYs?si=ILU_b3x879gxnLmM> | Question, keyterm | Kidney Transplant and What Foods to Eat and Avoid After, Blake Shusterman aka The Cooking Docs, 2022-Jul-25 | Hospital | USA | Patient (Adult) | No | 28,800 | 204 |
| 3 | <https://youtu.be/_QEyo00APpA?si=9LtsDN8sd_nI-o8e> | Question | What You Should and Shouldn’t Eat or Drink After Your Kidney Transplant, Cleveland Clinic, 2019-Nov-04 | Hospital | USA | Patient (Adult) | No | 16,626 | 129 |
| 4 | <https://youtu.be/e2-7Cp3KeLI?si=K7ooFIg8jgB33QXt> | Question | 6 Things You Should Know About Life After Kidney Transplant Surgery, Crozer Health, 2018-Nov-15 | Hospital | USA | Patients (adult and pediatric) | No | 948 | 23 |
| 5 | <https://www.youtube.com/live/6uoA2n1ULFw?si=LA3S_TXWR0hD-y6d> | Question, keyterm | Care for Your New Kidney: What You Need to Know After Your Kidney Transplant, Cleveland Clinic, 2022-Dec-13 | Healthcare Organization | Canada | Patient (adult) | No | 6,814 | 89 |
| 6 | <https://youtu.be/ForAVE6Ng1U?si=aPmf-Z5akJSbIq44> | Question | Kidney Transplant Nutrition Webinar, The Centre for Living Organ Donation (University Health Network), 2021-Aug-19 | Independent (Healthcare Provider) | India | Patient (Adult) | No | 46,071 | 388 |
| 7 | <https://youtu.be/0uLRIG-26EM?si=y7Vebd6XmcXhYXRq> | Question, keyterm | What is the suggested diet chart for a Kidney Transplanted patient? - Dr. Sankaran Sundar, Doctors' Circle World's Largest Health Platform, 2017-Jul-28 | Hospital | USA | Caregivers and Patients (Adult and Pediatric) | No | 685 | 11 |
| 8 | <https://youtu.be/FDPhS1J3AhY?si=ZmYqog5Y3VtxmGih> | Question | Understanding Your Kidney Function After An Organ Transplant, Stanford Medicine Children's Health, 2021-Jul-15 | Independent (Healthcare Provider) | USA | Patient (Adult) | No | 971 | 54 |
| 9 | <https://youtu.be/DYim6YrTZvA?si=C29n64M_bo-LvFql> | Question, keyterm | Nutrition After Kidney Transplant,Michele Crosmer (RD) and Sean Hashmi (MD), 2023-Jul-12 | Hospital | USA | Patient (Adult) | No | 3,805 | 43 |
| 10 | <https://youtu.be/B5F-hR_2QY0?si=6HMFIkVmVfNDPDYW> | Question | CAMC Kidney Transplant Center: Post-Transplant Nutrition, Stephanie Brumfield (RD), Charleston Area Medical Center, 2021-Feb-23 | Hospital | USA | Patient (Adult) | No | 5,748 | 54 |
| 11 | <https://youtu.be/IlhqEf8EOCg?si=sVNv1eFtaRuR2wfB> | Question, keyterm | Nutrition After Transplant, Beth Israel Deaconess Medical Center (BIDMC), 2017-Nov-27 | Hospital | USA | Patient (Adult) | No | 47,096 | 447 |
| 12 | <https://youtu.be/L0tHKIEHXlI?si=3hmyPvkPRlsi91KY> | Question | Living With Your Transplant - Post-Transplant Nutrition, Beverley Borjas (RD), UCHealthCincinnati | Hospital | India | Patient (Adult) | No | 41,365 | 606 |
| 13 | <https://youtu.be/RzCdBVlcNLs?si=JZelfyZa-JprXBio> | Question | Care after Kidney Transplant, Rammohan S Bhat (MD), NARAYANA HEALTH, 2016-Apr-16 | Independent (Healthcare Provider) | India | Patient (Adult) | No | 8,637 | 261 |
| 14 | <https://youtu.be/1binO9q5BLo?si=tPuNV7s1gPgreukY> | Question | SEVEN MYTHS REGARDING DIET OF KIDNEY TRANSPLANT PATIENTS (ENGLISH), DR SANJEEV GULATI, Dr Sanjeev Gulati Kidney Clinic, 2021-May-02 | Healthcare Organization | USA | Patient (Adult) | No | 2,023 | 15 |
| 15 | <https://youtu.be/QADWpNcGNbI?si=V1saJxBU9lU-AV1j> | Keyterm | Webinar: Healthy weight pre and post-kidney transplant, Brent Miller (MD), kidneyfund, 2019-Jul-09 | Healthcare Organization | USA | Patient (Adult) | No | 1,874 | 11 |
| 16 | <https://youtu.be/C0z6CudidMU?si=GyEG1T_5DpEE4HDX> | Keyterm | Post Transplant Kidney Diet \| ESRD NCC, End Stage Renal Disease National Coordinating Center (ESRD NCC), 2020-Nov-09 | Hospital | USA | Patient (Adult) | No | 547 | 4 |
| 17 | <https://youtu.be/YQEAwTFytRo?si=JfyB5aDiDzMTHrig> | Keyterm | Kidney Transplant: The Importance of Nutrition, UT Southwestern Medical Center, 2023-Aug-07 | Hospital | USA | Patient (Adult) | No | 10,895 | 0 |
| 18 | <https://youtube.com/shorts/8vmfkLIgpKE?si=ljfc-7uV-8lF3yf7> | Keyterm | Tips for healthy eating after transplant video, UW Health, 2020-Jun-25 | Hospital | India | Patient (Adult) | No | 2,385 | 51 |
| 19 | <https://youtu.be/7drzdT4_Inc?si=h0G3eRyJ9DnMb0R2> | Keyterm | Diet after getting a transplant, Vishal Saxena (MD), 2023-Jul-09 | Independent | USA | Patient (Adult) | No | 2,678 | 35 |
| 20 | <https://youtu.be/cd9XciaS9Tc?si=-eSB0kldk_dWyKfv> | Keyterm | Nutrition After Kidney Transplant - How do you keep your kidneys healthy after a transplant? \|247nht,  247 Natural Health Tricks, 2020-Jan-25 | Hospital | USA | Patient (Adult) | No | 592 | 6 |
| Exercise | | | | | | | | | |
| 1 | <https://youtu.be/_QEyo00APpA?si=U_f4gz2lzs5imkBf> | Question, keyterm | 6 Things You Should Know About Life After Kidney Transplant Surgery, Crozer Health, 2018-Nov-15 | Hospital | United States | Patient (Adult) | No | 16,629 | 129 |
| 2 | <https://youtu.be/e2-7Cp3KeLI?si=Q1BkRe7B6B59qrdg> | Question | Care for Your New Kidney: What You Need to Know After Your Kidney Transplant, Cleveland Clinic, 2022-Dec-13 | Hospital | United States | Patient (Adult) | No | 949 | 23 |
| 3 | <https://youtu.be/yuEi5Yf5nR8?si=eL972fyRLiCxPjrb> | Question | Can competitive athletics continue after a Kidney Transplant?, Sankaran Sundar (MD), 2017-Jul-28 | Independent (Healthcare Providers) | India | Patient (Adult) | No | 1,957 | 23 |
| 4 | <https://youtu.be/UZtCTClox5k?si=fMR8ClVMMjHe60ok> | Question, keyterm | Life After Kidney Transplant, Viadam, 2021-Apr-20 | Healthcare Organization | India | Patient (Adult) | No | 53,501 | 334 |
| 5 | <https://youtu.be/hshjH9VxyLQ?si=HyLwybhJmgtCVdfL> | Question | Helping Your Child Manage Their Kidney Transplant \| Rare Disease \| Cystinosis, American Kidney Fund, 2021-Jul-28 | Healthcare Organization | United States | Caregiver (Pediatric) | No | 1,746 | 6 |
| 6 | <https://www.youtube.com/watch?v=oeF_eI145EU&pp=ygVaQXJlIHRoZXJlIGFueSBwaHlzaWNhbCBhY3Rpdml0aWVzIG15IGNoaWxkIHNob3VsZG7igJl0IGRvIGFmdGVyIHRoZWlyIGtpZG5leSB0cmFuc3BsYW50PyAg> | Question | After Kidney Transplantation Do's & Dont's I Patient Education, Suhas Bavikar, 2019-Mar-30 | Independent (Healthcare Providers) | India | Patient (Adult) | No | 25,338 | 330 |
| 7 | <https://www.youtube.com/watch?v=EpD56QVWgCc&pp=ygVaQXJlIHRoZXJlIGFueSBwaHlzaWNhbCBhY3Rpdml0aWVzIG15IGNoaWxkIHNob3VsZG7igJl0IGRvIGFmdGVyIHRoZWlyIGtpZG5leSB0cmFuc3BsYW50PyAg> | Question | Kidney Transplant Aftercare: What happens after you get a new kidney, Crissy Mac (patient), 2020-Apr-29 | Independent (Patient) | United States | Patient (Adult) | Yes | 2,273 | 81 |
| 8 | <https://www.youtube.com/watch?v=MbeP-1DFrYo&pp=ygUxUGVkaWF0cmljIGtpZG5leSB0cmFuc3BsYW50IGV4ZXJjaXNlIGxpbWl0YXRpb25zIA%3D%3D> | Keyterm | Seven Secrets to Surviving Long after Kidney Transplant, Sanjeev Gulati, 2021-Apr-30 | Independent (Healthcare Providers) | India | Patient (Adult) | No | 33,490 | 795 |
| 9 | <https://www.youtube.com/watch?v=dHivmpn7tt8&pp=ygUxUGVkaWF0cmljIGtpZG5leSB0cmFuc3BsYW50IGV4ZXJjaXNlIGxpbWl0YXRpb25zIA%3D%3D> | Keyterm | My Exercise Routine as a Kidney Transplant Patient, Cindy Flores, 2018-Nov-13 | Independent (Patient) | United States | Patient (Adult) | Yes | 13,688 | 330 |
| Travel | | | | | | | | | |
| 1 | <https://www.youtube.com/watch?v=F_zv-0KxAuk&pp=ygVRV2hhdCBzaG91bGQgSSBrbm93IGFib3V0IHRyYXZlbGxpbmcgd2l0aCBteSBjaGlsZCBhZnRlciB0aGVpciBraWRuZXkgdHJhbnNwbGFudD8g> | Question, keyterm | Traveling After A Kidney Transplant...MUST WATCH!!! \| Travel tips for kidney patients, Crissy Mac, 2022-Oct-19 | Independent (patient) | United States | Patient (Adult) | Yes | 762 | 31 |
| 2 | <https://www.youtube.com/watch?v=v5ASWIAT1s4&pp=ygVRV2hhdCBzaG91bGQgSSBrbm93IGFib3V0IHRyYXZlbGxpbmcgd2l0aCBteSBjaGlsZCBhZnRlciB0aGVpciBraWRuZXkgdHJhbnNwbGFudD8g> | Question | FAQ ABOUT TRAVELING WITH A KIDNEY TRANSPLANT!, Cindy Flores, 2019-Nov-19 | Independent (patient) | United States | Patient (Adult) | Yes | 1,955 | 71 |
| 3 | <https://www.youtube.com/watch?v=BvxXdqjlH6Q&pp=ygVRV2hhdCBzaG91bGQgSSBrbm93IGFib3V0IHRyYXZlbGxpbmcgd2l0aCBteSBjaGlsZCBhZnRlciB0aGVpciBraWRuZXkgdHJhbnNwbGFudD8g> | Question, keyterm | Travel After Transplantation, Leonardo Riella, 2018-Feb-04 | Hospital | United States | Patient (Adult) | No | 637 | 12 |
| 4 | <https://www.youtube.com/watch?v=UZtCTClox5k&pp=ygVRV2hhdCBzaG91bGQgSSBrbm93IGFib3V0IHRyYXZlbGxpbmcgd2l0aCBteSBjaGlsZCBhZnRlciB0aGVpciBraWRuZXkgdHJhbnNwbGFudD8g> | Question | Life After Kidney Transplant, Viadam, 2021-Apr-20 | Healthcare Organization | India | Patient (Adult) | No | 53,501 | 334 |
| 5 | <https://www.youtube.com/watch?v=vCDSBbpXKeQ&pp=ygVRV2hhdCBzaG91bGQgSSBrbm93IGFib3V0IHRyYXZlbGxpbmcgd2l0aCBteSBjaGlsZCBhZnRlciB0aGVpciBraWRuZXkgdHJhbnNwbGFudD8g> | Question | Can I Travel After Organ Transplant?, Transplant Helper, 2019-Feb-15 | Independent (patient) | United States | Patient (Adult) | Yes | 469 | 34 |
| 6 | <https://www.youtube.com/watch?v=bCsPrfmm_fs&pp=ygVRV2hhdCBzaG91bGQgSSBrbm93IGFib3V0IHRyYXZlbGxpbmcgd2l0aCBteSBjaGlsZCBhZnRlciB0aGVpciBraWRuZXkgdHJhbnNwbGFudD8g> | Question | 7 TIPS WHEN TRAVELING WITH A TRANSPLANT, Cindy Flores, 2018-Aug-25 | Independent (patient) | United States | Patient (Adult) | Yes | 2,001 | 98 |
| 7 | <https://www.youtube.com/watch?v=-gJdBksge1E&pp=ygVRV2hhdCBzaG91bGQgSSBrbm93IGFib3V0IHRyYXZlbGxpbmcgd2l0aCBteSBjaGlsZCBhZnRlciB0aGVpciBraWRuZXkgdHJhbnNwbGFudD8g> | Question, keyterm | TRAVELING IMMUNE COMPROMISE- My first trip after my Kidney transplant. A few days in Mexico., Wendy Copeland, 2022-Jul-17 | Independent (patient) | United States | Patient (Adult) | Yes | 701 | 23 |
| Mental Health | | | | | | | | | |
| 1 | <https://www.youtube.com/watch?v=PdX4VA2dNMs&pp=ygVQSG93IGRvIEkgdGFrZSBjYXJlIG9mIG15IGNoaWxk4oCZcyBtZW50YWwgaGVhbHRoIGFmdGVyIHRoZWlyIGtpZG5leSB0cmFuc3BsYW50PyA%3D> | Question, keyterm | Solid Organ Transplant's Impact on Mental Health: Explained \| Cincinnati Children's, Katherine Bedard-Thomas and Kristin Rich, 2021-Mar-21 | Hospital | United States | Caregiver (Pediatric) | No | 394 | 3 |
| 2 | <https://www.youtube.com/watch?v=hshjH9VxyLQ&pp=ygVQSG93IGRvIEkgdGFrZSBjYXJlIG9mIG15IGNoaWxk4oCZcyBtZW50YWwgaGVhbHRoIGFmdGVyIHRoZWlyIGtpZG5leSB0cmFuc3BsYW50PyA%3D> | Question | Helping Your Child Manage Their Kidney Transplant \| Rare Disease \| Cystinosis, American Kidney Fund, 2021-Jul-28 | Healthcare Organization | United States | Caregiver (Pediatric) | No | 1,746 | 6 |
| 3 | <https://www.youtube.com/watch?v=yeO9ScNU8Pk&pp=ygVQSG93IGRvIEkgdGFrZSBjYXJlIG9mIG15IGNoaWxk4oCZcyBtZW50YWwgaGVhbHRoIGFmdGVyIHRoZWlyIGtpZG5leSB0cmFuc3BsYW50PyA%3D> | Question | 10 Tips And Advice \| Life After A Kidney Transplant, Crissy Mac, 2021-Feb-07 | Independent (patient) | United States | Caregivers and Adult patients | Yes | 7,918 | 315 |
| 4 | <https://www.youtube.com/watch?v=UuYk0e3eyRI&pp=ygVQSG93IGRvIEkgdGFrZSBjYXJlIG9mIG15IGNoaWxk4oCZcyBtZW50YWwgaGVhbHRoIGFmdGVyIHRoZWlyIGtpZG5leSB0cmFuc3BsYW50PyA%3D> | Question | Webinar: Adjusting to life after kidney transplant, Rupi Sodhi (MD), 2017-Nov-27 | Healthcare Organization | United States | Caregivers and Adult patients | No | 7,675 | 65 |
| 5 | <https://www.youtube.com/watch?v=gxAFxn1Cy3M&pp=ygVQSG93IGRvIEkgdGFrZSBjYXJlIG9mIG15IGNoaWxk4oCZcyBtZW50YWwgaGVhbHRoIGFmdGVyIHRoZWlyIGtpZG5leSB0cmFuc3BsYW50PyA%3D> | Question | Depression After Kidney Transplant?, One Mo Kidney, 2020-Aug-04 | Independent (patient) | United States | Patient (Adult) | Yes | 432 | 28 |

**Supplement 7. Readability scores for objective 1 (Google search)**

| Topic | Automated Readability Index | Flesch Reading Ease | Gunning Fog | Flesch-Kincaid Grade Level | Coleman-Liau Index | SMOG Index | Linsear Write Formula | Forcast Readability Formula | Average Reading Level Consensus Calculator |
| --- | --- | --- | --- | --- | --- | --- | --- | --- | --- |
| Topic 1: Healthy Eating | | | | | | | | | |
| Resource #1 | 7.56 | 71 | 8.6 | 5.9 | 9.92 | 6.43 | 5.03 | 11.02 | 8 |
| Resource #2 | 7.67 | 66 | 8.2 | 6.52 | 10.47 | 6.31 | 5.13 | 11.01 | 8 |
| Resource #3 | 7.25 | 63 | 9.5 | 6.63 | 10.35 | 6.91 | 4.6 | 10.98 | 8 |
| Resource #4 | 7.93 | 60 | 9.5 | 6.99 | 11.09 | 6.9 | 4.62 | 11.3 | 8 |
| Resource #5 | 7.08 | 71 | 8.6 | 6.24 | 9.2 | 6.53 | 5.9 | 10.37 | 8 |
| Resource #6 | 5.91 | 76 | 7.1 | 4.65 | 8.62 | 5.5 | 3.76 | 10.56/ N/A | 6* |
| Resource #7 | 8.97 | 60 | 10.9 | 8.66 | 10.2 | 8.03 | 8.42 | 10.5 | 9 |
| Resource #9 | 6.87 | 73 | 8.7 | 6.27 | 8.63 | 6.44 | 6.48 | 9.86/ N/A | 7* |
| Resource #10 | 9.08 | 54 | 10.9 | 8.35 | 12.24 | 7.91 | 6.1 | 11.45 | 9 |
| Resource #11 | 8.91 | 66 | 9.5 | 7.45 | 10.53 | 7.07 | 7.17 | 10.8 | 9 |
| Resource #12 | 9.53 | 61 | 10.3 | 7.9 | 11.7 | 7.55 | 6.95 | 10.87 | 9 |
| Resource #13 | 10.64 | 53 | 12.1 | 9.68 | 12.45 | 8.96 | 8.94 | 11.34 | 11 |
| Resource #14 | 7.03 | 72 | 8 | 5.64 | 9.56 | 6.04 | 4.68 | 10.68 | 7 |
| Resource #15 | 9.66 | 60 | 10.6 | 8.8 | 10.73 | 7.8 | 8.59 | 11.19 | 10 |
| Resource #16 | 6.91 | 70 | 8.5 | 6.05 | 9.33 | 6.46 | 5.3 | 10.19 N/A | 7* |
| Resource #17 | 5.83 | 75 | 7.7 | 4.91 | 8.35 | 5.9 | 4.05 | 11.43 / N/A | 6* |
| Resource #18 | 10.97 | 44 | 12.5 | 10.16 | 13.71 | 8.92 | 7.43 | 12.26 | 11 |
| Resource #19 | 9.52 | 62 | 10.8 | 8.15 | 11.1 | 8.02 | 7.93 | 10.39 | 9 |
| Resource #20 | 10.19 | 61 | 12.3 | 8.85 | 10.69 | 8.92 | 10.67 | 10 | 10 |
| Resource #21 | 7.2 | 69 | 8.7 | 6.21 | 9.75 | 6.54 | 5.35 | 10.36 | 8 |
| Resource #22 | 12.31 | 48 | 13 | 10.79 | 13.21 | 9.72 | 11.31 | 11.59 | 12 |
| Resource #23 | 11.01 | 48 | 12.8 | 10.21 | 12.8 | 9.7 | 10.17 | 12.16 | 11 |
| Resource #24 | 13.95 | 54 | 14.1 | 12.02 | 10.63 | 9.22 | 15.17 | 10.39 | 12 |
| Resource #25 | 7.71 | 62 | 10.2 | 7.27 | 10.54 | 7.42 | 5.65 | 11.42 | 9 |
| Resource #26 | 5.57 | 66 | 8.5 | 5.18 | 7.67 | 5.4 | 1.99 | 11.32 / N/A | 6* |
| Resource #27 | 5.61 | 68 | 8.5 | 5.92 | 8.47 | 6.39 | 4.4 | 10.46 / N/A | 7* |
| Resource #28 | 12.82 | 51 | 14.2 | 11.27 | 11.72 | 10.2 | 13.46 | 10.9 | 12 |
| Resource #29 | 8.74 | 56 | 10 | 7.98 | 12.29 | 7.24 | 5.63 | 11.95 | 9 |
| Resource #30 | 6.33 | 69 | 8 | 5.61 | 9.47 | 5.86 | 3.88 | 10.38 /N/A | 7* |
| Resource #31 | 8.12 | 68 | 9.7 | 6.9 | 10.16 | 7.29 | 6.76 | 9.62 | 8 |
| Resource #32 | 9.17 | 58 | 11 | 8.76 | 10.59 | 8.12 | 8.28 | 11.03 | 10 |
| Resource #33 | 8.76 | 69 | 10.2 | 7.61 | 9.18 | 7.24 | 8.5 | 9.57 | 9 |
| Resource #34 | 15.08 | 45 | 16.3 | 13.45 | 11.57 | 11.85 | 17.3 | 11.07 | 14 |
| Resource #35 | 6.98 | 69 | 8.8 | 5.9 | 9.89 | 6.5 | 4.64 | 10.48/ N/A | 7 |
| Topic 2: Exercise | | | | | | | | | |
| Resource #1 | 11.84 | 50 | 13.6 | 10.46 | 13.03 | 10.01 | 11.16 | 11.07 | 12 |
| Resource #2 | 10.13 | 62 | 12.5 | 9.35 | 9.4 | 8.9 | 11.87 | 9.82 | 10 |
| Resource #4 | 12.26 | 55 | 13.6 | 10.5 | 10.89 | 10.07 | 13 | 10.25 | 12 |
| Resource #5 | 6.93 | 65 | 10.9 | 6.95 | 9.19 | 7.88 | 6.3 | 10.56 | 8 |
| Resource #6 | 10.44 | 58 | 13.3 | 9.39 | 10.76 | 9.69 | 11.34 | 10.17 | 11 |
| Resource #7 | 14.68 | 33 | 17.4 | 13.35 | 15.14 | 12.48 | 14.17 | 11.84 | 14 |
| Resource #8 | 9.73 | 60 | 12.6 | 8.35 | 11.44 | 9.29 | 8.42 | 10.8 | 10 |
| Resource #9 | 5.09 | 81 | 7.5 | 4.74 | 6.81 | 5.73 | 5.38 | 9.6/ N/A | 6* |
| Resource #10 | 10.84 | 47 | 13.8 | 10.39 | 12.52 | 9.96 | 10.22 | 11.49 | 11 |
| Resource #11 | 7.28 | 65 | 9.6 | 6.32 | 10.34 | 6.77 | 4.33 | 11.65 | 8 |
| Resource #12 | 10.68 | 60 | 12.5 | 9.6 | 10.31 | 8.87 | 11.65 | 10.78 | 11 |
| Resource #13 | 11.3 | 48 | 12.6 | 9.76 | 13.72 | 9.26 | 7.91 | 12.04 | 11 |
| Resource #14 | 8.39 | 57 | 12.4 | 8.35 | 10.99 | 8.87 | 7.21 | 10.69 | 10 |
| Resource #15 | 14.5 | 42 | 15.8 | 12.34 | 14.4 | 11.4 | 13.81 | 11.66 | 13 |
| Resource #16 | 11.73 | 52 | 13.6 | 10.23 | 12.62 | 9.86 | 11.13 | 11.83 | 12 |
| Resource #17 | 7.59 | 64 | 10.1 | 6.83 | 10.45 | 7.33 | 5.39 | 10.96 | 8 |
| Resource #18 | 10.71 | 56 | 13 | 9.56 | 11.28 | 9.53 | 11 | 10.96 | 11 |
| Topic 3: Travel | | | | | | | | | |
| Resource #1 | 9.23 | 58 | 10.9 | 9.25 | 9.82 | 8 | 10.14 | 10.78 | 10 |
| Resource #2 | 12.53 | 48 | 14.6 | 11.28 | 12.66 | 10.59 | 12.67 | 11.05 | 12 |
| Resource #3 | 12.26 | 71 | 13.4 | 9.74 | 8.32 | 8.29 | 14.17 | 9.68 | 11 |
| Resource #4 | 3.58 | 78 | 6.4 | 4.17 | 6.3 | 5.06 | 3.2 | 9.66 / N/A | 5* |
| Resource #5 | 8.42 | 64 | 9.8 | 7.75 | 10.04 | 7.28 | 7.4 | 10.3 | 9 |
| Resource #6 | 14.97 | 48 | 16.6 | 13.11 | 11.37 | 11.57 | 17.25 | 10.24 | 14 |
| Resource #7 | 10.32 | 57 | 12.5 | 9.29 | 11.45 | 9.09 | 10.5 | 10.44 | 11 |
| Resource #8 | 7.38 | 68 | 9.2 | 6.89 | 9.38 | 6.77 | 6.4 | 10.6 | 8 |
| Resource #9 | 10.71 | 65 | 10.7 | 8.67 | 10.49 | 7.7 | 10.64 | 10.79 | 10 |
| Resource #10 | 9.11 | 68 | 11.3 | 8.49 | 8.67 | 8.04 | 11.06 | 9.82 | 10 |
| Resource #11 | 6.9 | 71 | 9.6 | 6.72 | 8.3 | 7.01 | 7.2 | 10/ N/A | 8* |
| Resource #12 | 12.13 | 47 | 13.9 | 11.29 | 12.22 | 10.07 | 12.33 | 10.89 | 12 |
| Resource #13 | 9.52 | 73 | 7.5 | 6.95 | 10.36 | 6.44 | 7.92 | 9.84 | 8 |
| Resource #14 | 9.1 | 62 | 10.3 | 8.68 | 9.87 | 7.6 | 8.83 | 10.51 | 9 |
| Resource #15 | 7.04 | 72 | 9 | 6.74 | 8.25 | 6.44 | 7.17 | 10.41 | 8 |
| Resource #16 | 14.11 | 42 | 16.7 | 13.22 | 12.41 | 12.48 | 16.25 | 11.26 | 14 |
| Resource #17 | 9.45 | 73 | 9.6 | 7.48 | 9.48 | 6.74 | 10.07 | 9.39 | 9 |
| Resource #18 | 7.11 | 61 | 10.9 | 7.53 | 9.52 | 8.05 | 6.55 | 10.51 | 9 |
| Resource #19 | 10.33 | 60 | 11.7 | 9.17 | 10.53 | 8.64 | 10.7 | 11.14 | 10 |
| Topic 4: Mental Health | | | | | | | | | |
| Resource #1 | 7.47 | 71 | 8.5 | 6.78 | 9.02 | 6.09 | 6.83 | 10.23 | 8 |
| Resource #2 | 9.33 | 57 | 11.8 | 8.75 | 11.29 | 8.64 | 8.05 | 11.06 | 10 |
| Resource #3 | 7.26 | 74 | 8.6 | 5.94 | 9.15 | 6.62 | 6.33 | 9.39 | 8 |
| Resource #4 | 12.96 | 41 | 13.6 | 11.15 | 15.23 | 9.87 | 10.24 | 12.08 | 12 |
| Resource #5 | 11.54 | 44 | 12.2 | 10.53 | 14.05 | 9.22 | 8.25 | 12.06 | 11 |
| Resource #6 | 6.03 | 79 | 7.5 | 5.04 | 7.94 | 5.59 | 5.47 | 9.43 / N/A | 6* |
| Resource #7 | 17.4 | 40 | 17.6 | 14.99 | 12.89 | 12.26 | 19.1 | 11.22 | 15 |
| Resource #8 | 8.63 | 61 | 11.1 | 8.23 | 10.22 | 8.17 | 7.92 | 10.39 | 9 |
| Resource #10 | 18.15 | 18 | 19.7 | 16.56 | 16.97 | 14.3 | 17.89 | 12.69 | 17 |
| Resource #11 | 10.69 | 61 | 12.2 | 9.34 | 10.65 | 8.79 | 11.53 | 9.86 | 10 |
| Resource #12 | 12.63 | 48 | 13.6 | 11 | 13.36 | 10.23 | 11.95 | 11.05 | 12 |
| Resource #13 | 9.19 | 64 | 9.1 | 7.69 | 11.13 | 6.77 | 7 | 10.6 | 9 |

**Supplement 8. PEMAT-P and PEMAT-AV Understandability and Actionability Scores**

| PEMAT-P Results: Health Eating Resources | | |
| --- | --- | --- |
| Resource # | Understandability Score | Actionability Score |
| 1 | 75% | 40% |
| 2 | 75% | 40% |
| 3 | 69% | 40% |
| 4 | 62% | 40% |
| 5 | 77% | 60% |
| 6 | 77% | 40% |
| 7 | 53% | 40% |
| 8 | 70% | 0% |
| 9 | 67% | 40% |
| 10 | 62% | 60% |
| 11 | 63% | 40% |
| 12 | 69% | 20% |
| 13 | 58% | 0% |
| 14 | 69% | 60% |
| 15 | 46% | 20% |
| 16 | 38% | 50% |
| 17 | 50% | 40% |
| 18 | 88% | 20% |
| 19 | 59% | 42% |
| 20 | 60% | 40% |
| 21 | 69% | 40% |
| 22 | 78% | 40% |
| 23 | 62% | 0% |
| 24 | 54% | 40% |
| 25 | 69% | 40% |
| 26 | 54% | 60% |
| 27 | 50% | 40% |
| 28 | 67% | 40% |
| 29 | 59% | 20% |
| 30 | 56% | 40% |
| 31 | 56% | 20% |
| 32 | 58% | 0% |
| 33 | 67% | 40% |
| 34 | 58% | 0% |
| 35 | 69% | 40% |
| PEMAT-P Results: Exercise | | |
| Resource # | Understandability Score | Actionability Score |
| 1 | 85% | 60% |
| 2 | 69% | 60% |
| 3 | 70% | 0% |
| 4 | 69% | 40% |
| 5 | 69% | 40% |
| 6 | 77% | 40% |
| 7 | 67% | 40% |
| 8 | 62% | 40% |
| 9 | 69% | 60% |
| 10 | 69% | 60% |
| 11 | 60% | 40% |
| 12 | 62% | 40% |
| 13 | 69% | 20% |
| 14 | 67% | 40% |
| 15 | 58% | 40% |
| 16 | 69% | 40% |
| 17 | 67% | 60% |
| 18 | 77% | 40% |
| PEMAT-P Results: Travel | | |
| Resource # | Understandability Score | Actionability Score |
| 1 | 69% | 40% |
| 2 | 69% | 40% |
| 3 | 85% | 40% |
| 4 | 67% | 40% |
| 5 | 63% | 40% |
| 6 | 58% | 60% |
| 7 | 67% | 40% |
| 8 | 67% | 40% |
| 9 | 69% | 40% |
| 10 | 69% | 60% |
| 11 | 69% | 40% |
| 12 | 75% | 40% |
| 13 | 75% | 40% |
| 14 | 58% | 40% |
| 15 | 75% | 40% |
| 16 | 67% | 40% |
| 17 | 69% | 40% |
| 18 | 63% | 60% |
| 19 | 38% | 40% |
| 20 | 75% | 100% |
| PEMAT-P: Mental Health | | |
| Resource # | Understandability Score | Actionability Score |
| 1 | 85% | 40% |
| 2 | 50% | 40% |
| 3 | 58% | 40% |
| 4 | 58% | 0% |
| 5 | 50% | 40% |
| 6 | 67% | 40% |
| 7 | 63% | 40% |
| 8 | 75% | 40% |
| 9 | 90% | 67% |
| 10 | 58% | 0% |
| 11 | 42% | 40% |
| 12 | 67% | 60% |
| 13 | 67% | 40% |
| 14 | 90% | 0% |
| PEMAT-AV: Healthy Eating | | |
| Resource # | Understandability Score | Actionability Score |
| 1 | 73% | 67% |
| 2 | 73% | 67% |
| 3 | 70% | 67% |
| 4 | 91% | 67% |
| 5 | 83% | 67% |
| 6 | 45% | 33% |
| 7 | 75% | 75% |
| 8 | 56% | 67% |
| 9 | 73% | 100% |
| 10 | 92% | 100% |
| 11 | 85% | 100% |
| 12 | 67% | 67% |
| 13 | 44% | 67% |
| 14 | 67% | 100% |
| 15 | 73% | 33% |
| 16 | 75% | 100% |
| 17 | 55% | 75% |
| 18 | 43% | 67% |
| 19 | 36% | 67% |
| 20 | 70% | 67% |
| PEMAT-AV: Exercise | | |
| Resource # | Understandability Score | Actionability Score |
| 1 | 70% | 67% |
| 2 | 82% | 100% |
| 3 | 55% | 67% |
| 4 | 46% | 33% |
| 5 | 73% | 67% |
| 6 | 27% | 100% |
| 7 | 60% | 67% |
| 8 | 44% | 67% |
| 9 | 56% | 100% |
| PEMAT-AV: Travel | | |
| Resource # | Understandability Score | Actionability Score |
| 1 | 91% | 100% |
| 2 | 78% | 100% |
| 3 | 73% | 100% |
| 4 | 45% | 33% |
| 5 | 78% | 100% |
| 6 | 90% | 100% |
| 7 | 67% | 67% |
| PEMAT-AV: Mental Health | | |
| Resource # | Understandability Score | Actionability Score |
| 1 | 55% | 0% |
| 2 | 73% | 100% |
| 3 | 91% | 100% |
| 4 | 69% | 100% |
| 5 | 56% | 67% |
