# Supplementary material for: How Well Do “Dr. Google” and “Professor YouTube” Answer Health Questions for Pediatric Kidney Transplant Recipients and Caregivers?
Source: Pediatr Transplant. 2026 Aug 11;30(8):e70409. doi: 10.1111/petr.70409 (PMC13458959; doi:10.1111/petr.70409)
Supplement: Supplementary file 2 [file PETR-30-e70409-s002.docx]

Appendix 1: CST Pediatric Education initiative List of Education topics

1. What are kidneys and what do they do?

2. What is a kidney transplant and how will it help me?

3. What are the types of kidney transplant?

4. ABO blood types and human leukocyte antigen (HLA)

5. What do I need to do to get ready for a kidney transplant?

6. What will it mean when I have a kidney transplant?

7. How will medicines help in kidney transplant?

8. What are the Infection risks in kidney transplant? Can they be prevented or treated?

9. What does rejection mean in kidney transplant? Can it be prevented or treated?

10. What is Adherence and what does it mean for a kidney transplant?

11. How can I help my kidney transplant last as long as possible?

12. Will I need a second transplant and, what can I do to keep my first kidney as long as possible

13. Dental Care

14. Fluids

15. Healthy eating

16. Skin Care

17. Sexuality

18. Exercise

19. Pets

20. Travel

21. Safe Living

22. Post-transplant Lymphoproliferative Disease (PTLD) and malignancy

23. Alcohol and cannabis

24. Mental Health

25. School
